# CRISPR/Cas9 Genome Engineering in Engraftable Human Brain-Derived Neural Stem Cells

**DOI:** 10.1016/j.isci.2019.04.036

**Published:** 2019-05-04

**Authors:** Daniel P. Dever, Samantha G. Scharenberg, Joab Camarena, Eric J. Kildebeck, Joseph T. Clark, Renata M. Martin, Rasmus O. Bak, Yuming Tang, Monika Dohse, Johannes A. Birgmeier, Karthik A. Jagadeesh, Gill Bejerano, Ann Tsukamoto, Natalia Gomez-Ospina, Nobuko Uchida, Matthew H. Porteus

**Affiliations:** 1 Department of Pediatrics, Stanford University, Stanford, CA 94305, USA; 2 Department of Developmental Biology, Stanford University, Stanford, CA 94305, USA; 3 Department of Computer Science, Stanford University, Stanford, CA 94305, USA; 4 StemCells Inc, Newark, CA 94560, USA; 5 ReGen Med Division, BOCO Silicon Valley, Palo Alto, CA 94303, USA

**Keywords:** Molecular Biology, Neuroscience, Bioengineering, Biotechnology, Cell Biology, Biological Sciences Research Methodologies

## Abstract

Human neural stem cells (NSCs) offer therapeutic potential for neurodegenerative diseases, such as inherited monogenic nervous system disorders, and neural injuries. Gene editing in NSCs (GE-NSCs) could enhance their therapeutic potential. We show that NSCs are amenable to gene targeting at multiple loci using Cas9 mRNA with synthetic chemically modified guide RNAs along with DNA donor templates. Transplantation of GE-NSC into oligodendrocyte mutant shiverer-immunodeficient mice showed that GE-NSCs migrate and differentiate into astrocytes, neurons, and myelin-producing oligodendrocytes, highlighting the fact that GE-NSCs retain their NSC characteristics of self-renewal and site-specific global migration and differentiation. To show the therapeutic potential of GE-NSCs, we generated GALC lysosomal enzyme overexpressing GE-NSCs that are able to cross-correct GALC enzyme activity through the mannose-6-phosphate receptor pathway. These GE-NSCs have the potential to be an investigational cell and gene therapy for a range of neurodegenerative disorders and injuries of the central nervous system, including lysosomal storage disorders.

## Highlights

• Human neural stem cells are amenable to CRISPR-Cas9-mediated gene targeting
• Truncated CD19 surface marker allows for enrichment of gene-targeted NSCs to >90%
• Gene-targeted NSCs engraft, migrate, and differentiate in immunodeficient mice
• GALC-engineered overexpressing NSCs cross-correct Krabbe disease fibroblasts *in vitro*

## Graphical Abstract

**Figure:**
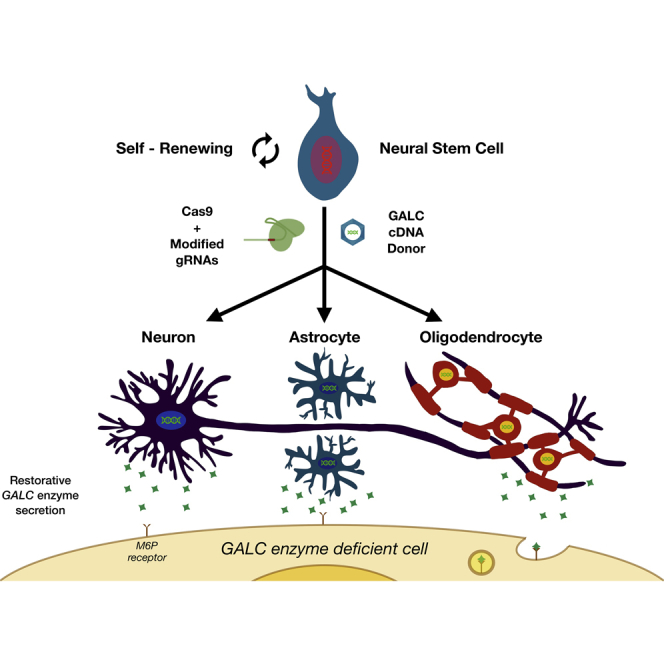

## Introduction

Human neural stem cells (NSCs) are self-renewing, multilineage-producing cells that hold great promise for treating a large number of central nervous system (CNS) disorders. One specific NSC identified has demonstrated self-renewal at the single-cell level (Uchida et al., 2000). Based on these initial characterizations, individual NSC banks (to distinguish from freshly isolated NSCs) have been successfully established from single donated human fetal brain tissue (16–20 weeks' gestation) by directly isolating CD133^+^/CD24^-/lo^ NSCs with selective propagation in a defined serum-free culture media grown as neurospheres and cryopreserved (and therefore banked). Previous studies have shown that these NSCs are capable of proliferation, migration, and multilineage differentiation in a site-appropriate manner when they were transplanted into brains or spinal cord of immunodeficient mice, thereby recapitulating temporal development stage of NSCs from the human fetal brain (Uchida et al., 2012). No genetic modification or immortalization was required, and these banked cells continue to express NSC markers such as CD133 and Sox2 (Carpenter et al., 1999, Cummings et al., 2005, Tamaki et al., 2002, Tamaki et al., 2009, Tsukamoto et al., 2013, Uchida et al., 2000, Uchida et al., 2012). They have biological NSC activities with multiple mechanisms of actions, providing neuroprotection, myelination, and retinal preservation via site-appropriate global migration.

Banked NSCs have been transplanted into a mouse model of lysosomal storage disorder (LSD) with PPT1 deficiency (also known as infantile neuronal ceroid lipofuscinosis [NCL] or Batten disease), and it has been demonstrated that these cells engrafted and migrated extensively, which led to reduced accumulated toxic storage material and ultimately broad neuroprotection of host cells in the hippocampus and cortex (Tamaki et al., 2009). These preclinical studies led to the first-in-human NSC transplantation for infantile and late-infantile NCLs authorized by the US Food and Drug Administration (Selden et al., 2013). Banked NSCs can also induce functional myelin in a severe demyelination model (Uchida et al., 2012), the shiverer mouse, and restore motor function in a mouse model of spinal cord injury ( Cummings et al., 2005, Salazar et al., 2010). The proof-of-concept data in pre-clinical studies led to other phase I or II studies with NSCs in the hypomyelinating disease, Pelizaeus-Merzbacher disease (PMD) (Gupta et al., 2012), traumatic thoracic and cervical spinal cord injury (Levi et al., 2017), and age-related macular degeneration (clinical trial number NCT01632527). These clinical studies demonstrated the safety, feasibility, long-term survival, and early evidence of efficacy of using banked NSCs in treating neurologic diseases.

One approach to increasing the therapeutic efficacy of banked NSCs would be to enhance their potential through genome engineering technologies (Porteus, 2016). Recently, transcription activator-like effector nucleases (TALENs) (Boch et al., 2009) and RNA-guided endonucleases of the CRISPR/Cas9 system (CRISPRs) have both been engineered to create site-specific DSBs in human cells. In particular, the CRISPR/Cas9 platform allows for ease, versatility, and efficacy of creating locus-specific DSBs in human cells (Jinek et al., 2012, Cong et al., 2013). The CRISPR system (derived from *Streptococcus pyogenes*) uses RNA-DNA Watson-Crick hybridization to identify the DNA target site to be cleaved. CRISPR deploys two components to create DSBs: (1) a 100-nucleotide (nt) RNA guide (single guide RNA [sgRNA]) of which 20 nt recognize the target site and (2) the Cas9 protein molecule that cleaves both DNA strands creating a DNA double-strand break (DSB). The resulting DSB can be resolved by one of the two highly conserved repair mechanisms, canonical non-homologous end joining (NHEJ) or homologous recombination (HR) (Kass and Jasin, 2010, Porteus, 2016). Canonical NHEJ repair functions throughout the cell cycle to correct erroneous breaks through ligation of DNA without end processing, usually resulting in small insertions or deletions (INDELs). On the contrary, HR occurs specifically during S or G2 phase of the cell cycle when an undamaged sister chromatid is available. If the cell uses HR to repair a DSB with an exogenous donor template, then precise nucleotide changes to the genome can be introduced (genome editing).

Genome editing of human neuronal-derived lineages has been mostly accomplished through embryonic stem cell (ESCs) or induced pluripotent stem cell (iPSCs) methodologies (Heidenreich and Zhang, 2016, Gaj et al., 2017). Although iPSCs allow for neuronal disease modeling, like Parkinson and Alzheimer diseases, and also functional genomic analyses during formation of complex neuronal circuits, their clinical use for CNS disorders has been limited thus far because of safety concerns (Ben-David and Benvenisty, 2011). Recently, the first CRISPR/Cas9 gene targeting was shown in NSC mammalian cell lines. This article highlighted that CRISPR/Cas9 can be used to edit safe harbor loci *AAVS1* and *Rosa26*, biallelically knock out as well as epitope tag essential neuronal transcription factors, and engineer in glioma-initiating mutations. However, assessment of NSC function and multilineage potential *in vivo* as well as engineering NSCs for therapeutic value was missing (Bressan et al., 2017). The banked NSCs on the other hand have been extensively characterized *in vivo* and also have been used in multiple clinical trials, making genome editing of somatically derived NSCs a more feasible clinical approach for CNS disorders in the near future.

Here, we describe proof-of-concept genome editing studies in human-brain-derived propagated and banked NSCs that demonstrate the following: (1) genome editing in NSCs (GE-NSC) at three (*IL2RG*, *HBB*, *CCR5*) loci; (2) development of a simplified and robust cell surface marker enrichment scheme to generate a population of GE-NSCs using magnetic microbead technologies with greater than 90% purity; (3) GE-NSCs display robust engraftment and maintain biological NSC properties showing migration, differentiation, and long-term self-renewal and survival *in vivo*; and (4) generation of GALC lysosomal enzyme producing GE-NSCs that can cross-correct GALC enzyme activity in fibroblasts from patients affected with Krabbe disease. Collectively, these studies set the foundation for engineering and manufacturing human NSCs using genome editing to increase therapeutic potency of these cells to treat CNS disorders.

## Results

### Engineered Nuclease (CRISPR/Cas9 and TALEN)-Mediated Homologous Recombination in NSCs

**Figure 1:**
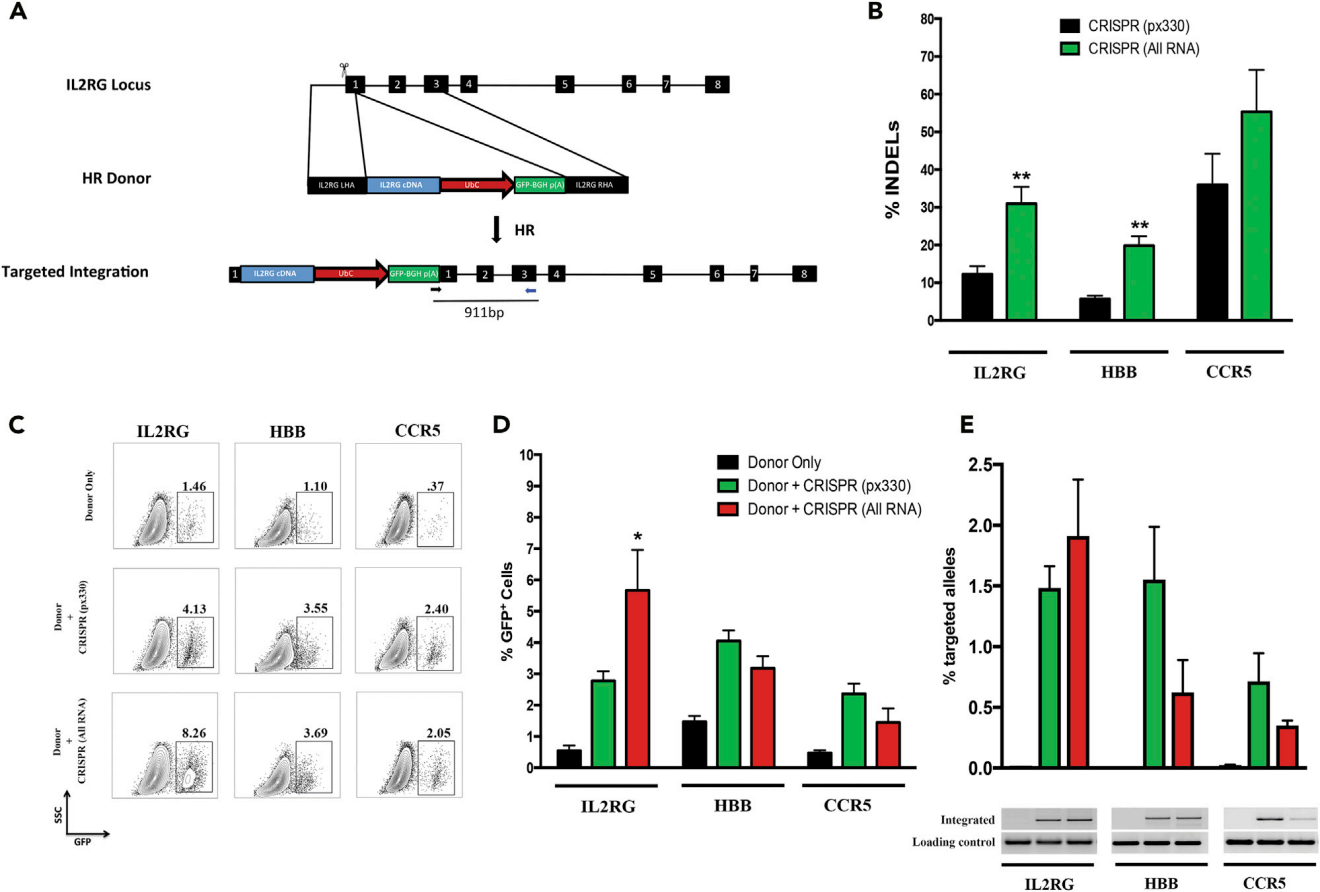
CRISPR/Cas9-Mediated Homologous Recombination (HR) in NSCs (A) The IL2RG locus was targeted for homologous recombination (HR) by creating double-strand breaks (DSBs) using Cas9 (scissors) and supplying a homologous donor template (with flanking 800-bp arms around the transgene to be inserted). Following HR, *IL2RG* cDNA is knocked in to the endogenous start codon with a UbC promoter driving GFP downstream to assess efficiencies of HR in human NSCs. *IL2RG* exons are shown in black boxes. (B) NSCs were electroporated with 1-μg plasmid (px330) encoding Cas9 and sgRNA specific for *IL2RG*, *HBB*, or *CCR5*. For “All RNA” experiments, Cas9 was delivered as mRNA (5 μg), and the locus-specific sgRNAs (10 μg) were delivered with terminal chemical modifications comprising 2′-*O*-methyl 3′phosphorothioate (MS) or 2′-*O*-methyl 3′thioPACE (MSP). Following 7 days in culture, gDNA was harvested, PCR was carried out to amplify the region of interest, and INDELs were calculated using the Tracking of Indels by Decomposition (TIDE) software. (N = 2–14), **p < 0.01, Student's t test. (C) NSCs were nucleofected as described above with either 2 μg HR donor or 2 μg HR and locus-specific CRISPR components. Locus-specific HR donor plasmids have at least 400 bp of homology flanking UbC-GFP to assess HR efficiencies. Cells were grown for at least 30 days until episomal HR donor was lost by dilution during proliferation to confirm stable expression of integrated cassettes into the *IL2RG*, *HBB*, and *CCR5* loci. Representative fluorescence-associated cell sorting plots are shown, which highlight stable integration of HR donors with site-specific nucleases. (D) HR frequencies obtained from all experiments targeting *IL2RG*, *HBB*, and *CCR5* carried out as above. (N = 3–7), *p < 0.05, Student's t test. (E) gDNA was harvested from experimental groups at day 30 post-culture to evaluate site-specific integration of HR donors on the DNA level. Digital droplet PCR (ddPCR) was used to quantify allelic integration of HR donors, (N = 3–4). Agarose gel electrophoresis of in-out PCR products confirms on-target integration of HR donors in the presence of a site-specific nuclease. Data are represented as mean ± SEM.

We first compared genome-editing efficiencies at the NSC “safe harbor” locus, interleukin-2 receptor gamma chain (*IL2RG*), in NSCs using *IL2RG*-specific TALEN pairs and the CRISPR/Cas9 system. The *IL2RG* locus is a safe harbor for transgene expression in NSCs because it is not expressed in NSCs or any NCS progeny and people with mutations in the *IL2RG,* although have a devastating severe combined immunodeficiency (SCID-X1) have no neurologic problems. To characterize the frequency of DSBs induced, we electroporated plasmids encoding either optimized *IL2RG*-specific TALEN pairs or CRISPR components (Cas9 and sgRNA), cultured cells for 7 days, isolated genomic DNA (gDNA), and then analyzed INDEL frequencies. The CRISPR system generated an average of 15% INDELs compared with 5% using the TALEN (Figure S1A, Related to Figure 1). We next compared mRNA delivery of *IL2RG* TALEN pairs to mRNA delivery of Cas9 along with chemically modified sgRNAs (“All RNA”). Electroporating NSCs with the All RNA CRISPR system induced 5-fold more INDELs than mRNA delivery of TALENs (Figure S1B, related to Figure 1). As both engineered nuclease systems were inducing DSBs (albeit at different frequencies), we targeted NSCs for HR with a plasmid encoding a homologous *IL2RG* cDNA-UbC-GFP donor cassette (Figure 1A), or a plasmid that introduced single nucleotide changes. Targeted NSCs were cultured for at least 30 days to allow episomal donor expression to dilute out such that fluorescence-activated cell sorting analysis would measure the frequency of cells with cassettes stably integrated by HR. Plasmid-based delivery of both engineered nuclease platforms mediated stable GFP expression in an average of 3.5% of NSCs (Figure S1C, related to Figure 1), whereas by using restriction fragment-length polymorphism to analyze the frequency of targeted alleles, the CRISPR system consistently modified more *IL2RG* alleles (Figure S1D, related to Figure 1). These data suggested that the CRISPR system was superior to TALENs, and therefore, was the nuclease used for the remainder of the studies described. These data also show that the *IL2RG* locus is an effective safe harbor in NSCs not only because the gene plays no functional role in these cells but also because that it is permissive for high-level transgene expression when a donor cassette using a ubiquitous promoter is inserted into the locus, while presumably not affecting the expression of neighboring NSC genes.

We next tested whether the CRISPR system could edit two other NSC “safe harbor” loci, the chemokine (C-C motif) receptor 5 (*CCR5*) and β-globin (*HBB*) genes. Both genes are not expressed in NSCs or their progeny, and people with mutations in these genes have no neurologic consequences from those mutations and thus qualify as safe harbors in NSCs. Plasmids encoding Cas9 and sgRNAs specific to *IL2RG*, *HBB*, and *CCR5* were electroporated into NSCs, and 7 days later, gDNA was extracted and alleles were analyzed for INDEL frequencies. The CRISPR-Cas9 system induced an average of 12%, 6%, and 36% INDELs at the *IL2RG*, *HBB*, and *CCR5* loci, respectively (Figure 1B). Consistent with our previous reports, delivery of Cas9 as mRNA and chemically modified sgRNAs induced more INDELs than plasmid delivery at all three loci tested (Figure 1B). To see if DSB formation promoted HR at all three loci, NSCs were co-electroporated with homologous DNA donor templates encoding GFP, and corresponding CRISPR components delivered as plasmid or All RNA. Our results showed that all three loci were amenable to HR in NSCs using plasmid or All RNA delivery of CRISPR-Cas9 components (Figures 1C and S2, related to Figure 1). Targeting NSCs at *IL2RG*, *HBB*, and *CCR5* loci with plasmid delivery of CRISPR resulted in an average of 2.8%, 4.1%, and 2.4% GFP^+^ NSCs after at least 30 days in culture, respectively (Figure 1D). Interestingly, All RNA delivery of *IL2RG* CRISPR resulted in 2-fold more GFP^+^ NSCs than plasmid delivery. To determine if HR occurred at the intended loci, gDNA was analyzed for on-target integration of the donor construct by in-out digital droplet PCR (ddPCR) (where one primer binds outside the homology arm of the donor construct and the other primer binds inside the donor cassette), thus only on-target integration events can be PCR quantified. PCR amplification was not detected with mock or donor only samples, whereas on-target integration was evident when NSCs were co-electroporated with CRISPR and a homologous donor, confirming HR at the intended locus (Figure 1E). As seen before, All RNA delivery of IL2RG CRISPR increased the frequency of targeted alleles, whereas HBB and CCR5 All RNA CRISPR delivery mediated fewer on-target integrations. Of note, we report an under-representation of on-target alleles compared with GFP+ cells for targeting *CCR5* and *HBB*, which may be due to non-target integrations of the donor-plasmid DNA, incomplete HR that interrupted ddPCR primer or probe sequences in the bovine growth hormone polyadenylation (BGH) poly(A) region, or the fact that there is still some episomal DNA expressing GFP after 30 days in culture in CRISPR-treated human NSCs. Because Cas9 can create DSBs at unintended genomic locations (Wu et al., 2014), we investigated off-target DSB activity at three predicted *IL2RG* off-target sites that we have previously assayed by next-generation sequencing in K562 cells (Hendel et al., 2015). We detected 28% DSB activity at *IL2RG*, whereas less than 0.7% cleavage was seen at any of the off-target sites investigated (Table 1). Collectively, these studies demonstrated that CRISPR-Cas9 can mediate on-target HR at multiple “safe harbor” loci in NSCs with minimal off-target cleavage. Thus these data establish that all three loci can be effective safe harbors in human NSCs as they support transgene expression from a cassette using an endogenous ubiquitous promoter. These data also provide further evidence that HR-mediated genome editing can be active at loci that are not expressed (Cameron et al., 2017) and that even though these loci are not naturally transcriptionally active in NSCs, they can still support transgene expression when an expression cassette is inserted into them. The level of transgene expression at the *HBB* locus is lower than at the other two loci, which might be used to tailor expression levels in certain applications.

**Table 1:** Low Off-Target Activity of *IL2RG* CRISPR/Cas9 System in Human NSCs

	Unselected	CD8 Selected	CD8 Selected	tCD19 Selected
IL2RG	28.35	75.85	59.11	71.27
Off-target 1	0	0	0.01	0
Off-target 2	0	0.28	0.07	0
Off-target 3	0	0	0	0.62

Targeted deep sequencing of *IL2RG* alleles and three previously predicted off-target sites was performed on unedited NSCs and GE-NSCs. Each column represents an independent targeting experiment from GE-NSCs that were unselected, CD8 selected, or tCD19 selected. Off-targets were previously predicted using bioinformatic tools (Hendel et al., 2015). All numbers are subtracted from background INDEL frequencies in an unedited NSC sample.

### Enrichment of Genome-Edited NSCs (GE-NSCs) by Magnetic-Activated Cell Sorting (MACS) Selection

**Figure 2:**
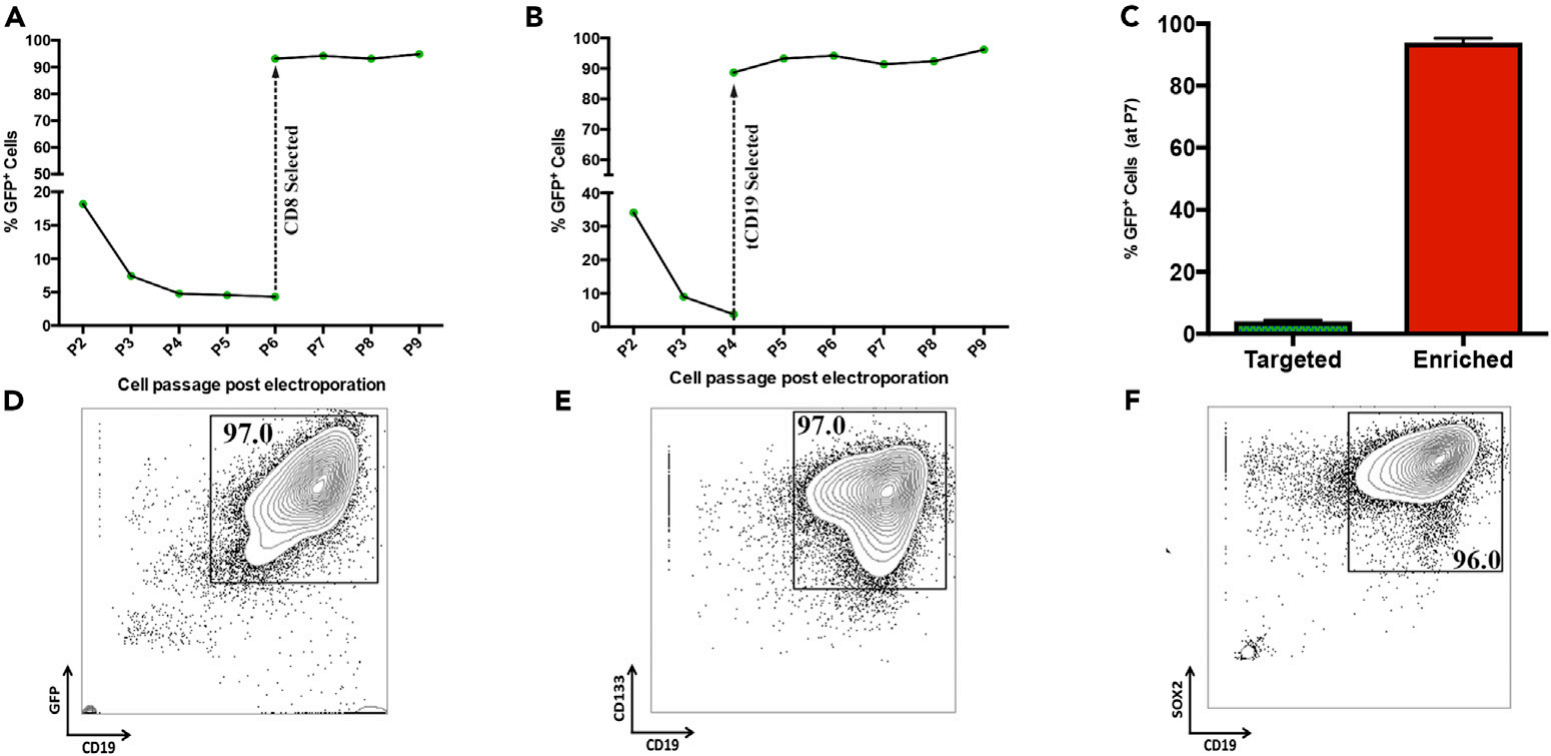
Enrichment of Genome-Edited NSCs (GE-NSCs) by Magnetic-Activated Cell Sorting (MACS) Selection A bicistronic *IL2RG* HR cassette was created that separates GFP from CD8 or truncated CD19 (tCD19) via a T2A peptide motif to allow robust magnetic bead enrichment of *IL2RG*-targeted NSCs. NSCs were nucleofected with 2 μg HR donor and 1 μg plasmid encoding Cas9 and sgRNA. Cells were grown for 30 days to allow episomal HR donor to dilute out during proliferation of NSCs. (A) GE-NSCs were cultured for nine passages post-electroporation while being analyzed for GFP expression at every cell passage. Cells were selected using CD8 microbead technologies at passage 6 post-targeting. (B) GE-NSCs were cultured for nine passages while being analyzed for GFP expression. (C) Frequencies of GE-NSCs using the bicistronic donor cassettes before (targeted) and after MACS selection (enriched), N = 3, Data are represented as mean ± SEM. (D–F) Expanded/MACS-enriched GE-NSCs with bicistronic GFP-T2a-tCD19 cassette were enzymatically dissociated into a single cell suspension and then stained for the cell surface markers, CD133 (Phycoerythrin [PE]) and CD19 (Allophycocyanin [APC]), and analyzed by FACS; (D) CD19 versus GFP and (E) CD19 versus CD133. (F) Cells were stained for CD19 (APC), permeabilized, fixed, and then stained again for SOX2 (PE).

Enrichment and expansion of genome-edited NSCs (GE-NSCs) to clinically relevant transplantable cell numbers would greatly increase their therapeutic potential. We devised an enrichment paradigm by establishing expression of the alpha chain of the CD8 protein dimer (CD8α) following successful HR at *IL2RG* coupled with a one-step magnetic-activated cell sorting (MACS) enrichment. CD8 complex requires dimerization of alpha and beta chains to transduce intercellular signals in cytotoxic T cells. Of note, expression of CD8α chain only has been reported not to be functional and should not alter NSC biologic function. In contrast, the truncated nerve growth factor (NGF) receptor might be predicted to perturb the natural signaling pathways of the brain by serving to “steal” NGF from cells that need NGF for their function. Thus the CD8 cell surface molecule on these cells can be used as a biologically inert cell surface marker to enrich for genetically modified NSCs. We therefore created an *IL2RG* HR donor construct (*IL2RG* cDNA-UbC-GFP-T2A-CD8α) that upon successful targeting would express a multigene mRNA, but two distinct GFP and CD8α proteins. NSCs were electroporated with a plasmid encoding Cas9 and sgRNA specific to *IL2RG* and also the CD8α HR donor cassette described above. Six cell passages post-electroporation, 4.7% of NSCs were stably expressing GFP (Figure S3A, left, related to Figure 2), similar to what we found with the GFP-only construct (Figure 1C). Then NSCs were subjected to a one-step CD8α magnetic bead enrichment protocol that resulted in >90% of cells expressing GFP (Figure S3A, right, related to Figure 2), and these cells were shown to expand and continually express GFP for multiple passages post-selection, confirming they were a population of GE-NSCs (Figure 2A).

In addition to CD8α, we also evaluated CD19 cell surface protein for our MACS-based enrichment protocol. Unlike the CD8 complex, CD19 is a single-chain molecule, and functional CD19 has cytoplasmic phosphorylated tyrosine domain, which transduces a signal (Depoil et al., 2008). Therefore we created a truncated form of the CD19 cell surface protein, where the intracellular signaling domain is removed, thus making CD19 signaling inert and solely serving as a cell surface marker for enrichment. In addition, even full-length CD19 has no known role in neurogenesis or gliogenesis and is not known to be expressed in any neural lineage. We therefore created an *IL2RG* HR donor construct (*IL2RG* cDNA-UbC-GFP-T2A-tCD19) and targeted NSCs for HR at the *IL2RG* locus. At passage four post-electroporation, 3.8% of NSCs were stably expressing GFP and cells were then subjected to MACS-based enrichment of tCD19 (Figure S3B, left, related to Figure 2). tCD19 selection resulted in enrichment and expansion of >90% of GE-NSCs, similar to CD8 selection of GE-NSCs (Figures 2A–2C). Using this enrichment methodology, it is also possible to select for cells with biallelic integration using two DNA donor molecules, each with a different fluorochrome (Bak et al., 2017). To test this in NSCs, we targeted the *CCR5* locus with both UbC-GFP and UbC-mCherry-T2A-tCD19 constructs. Targeting with both constructs resulted in 0.19% cells with targeted integration at both alleles, and CD19 MACS enrichment resulted in an ∼100-fold increase (12%) of biallelically targeted NSCs (Figure S4, related to Figure 2). Because we are enriching cells with HR events, we next wanted to investigate whether we were also selecting GE-NSCs with a higher frequency of off-target INDELs. Although we observed a 3-fold enrichment of *IL2RG* INDELs (because the banked NSCs are female with two X chromosomes), off-target INDELs were all less than 1% in CD8- and tCD19-selected GE-NSCs (Table 1).

To determine if GE-NSCs maintained their NSC characteristics, we enriched *IL2RG*-targeted NSCs to over 95% (Figure 2D) via the tCD19 selection scheme and then analyzed the expression of the two quintessential NSC markers: the cell surface gene CD133 (Figure 2E) and the intracellular NSC-maintaining transcription factor SOX2 (Figure 2F) (Hook et al., 2011). Following four weeks of expansion post-enrichment, greater than 95% of NSCs were CD133/CD19/GFP/SOX2 positive, highlighting that targeted neurospheres may fully possess NSC potential. These data provide two separate examples of the feasibility of a simplified one-step MACS-based protocol for enriching and then expanding >90% of GE-NSCs for transplantation.

Although our MACS-based enrichment strategy consistently resulted in purification to >90% of GE-NSCs, we also tested the recombinant adeno-associated virus serotype six (rAAV6) homologous donor template platform that has been shown to work well in primary cells (Dever et al., 2016, Sather et al., 2015, Wang et al., 2015). We electroporated Cas9 mRNA and *HBB* MSP sgRNAs into NSCs and then immediately transduced them with rAAV6 (Bak et al., 2018) carrying a UbC-GFP cassette with arms of homology for *HBB*. Using vector genomes per cell of 10,000, 100,000, and 500,000, we were able to achieve mean allelic targeting frequencies of 1.26%, 9.93%, and 12.57%, respectively (Figure S5, related to Figure 2). These results show that AAV6 is a highly active homologous donor in human NSCs that warrants future investigation. Given the enrichment to >90% purity using the one-step magnetic bead purification from using the plasmid donor, we pursued further *in vivo* analysis using these GE-NSCs.

### GE-NSCs Migrate and Differentiate into Neurons, Astrocytes, and Oligodendrocytes *In Vivo*

**Figure 3:**
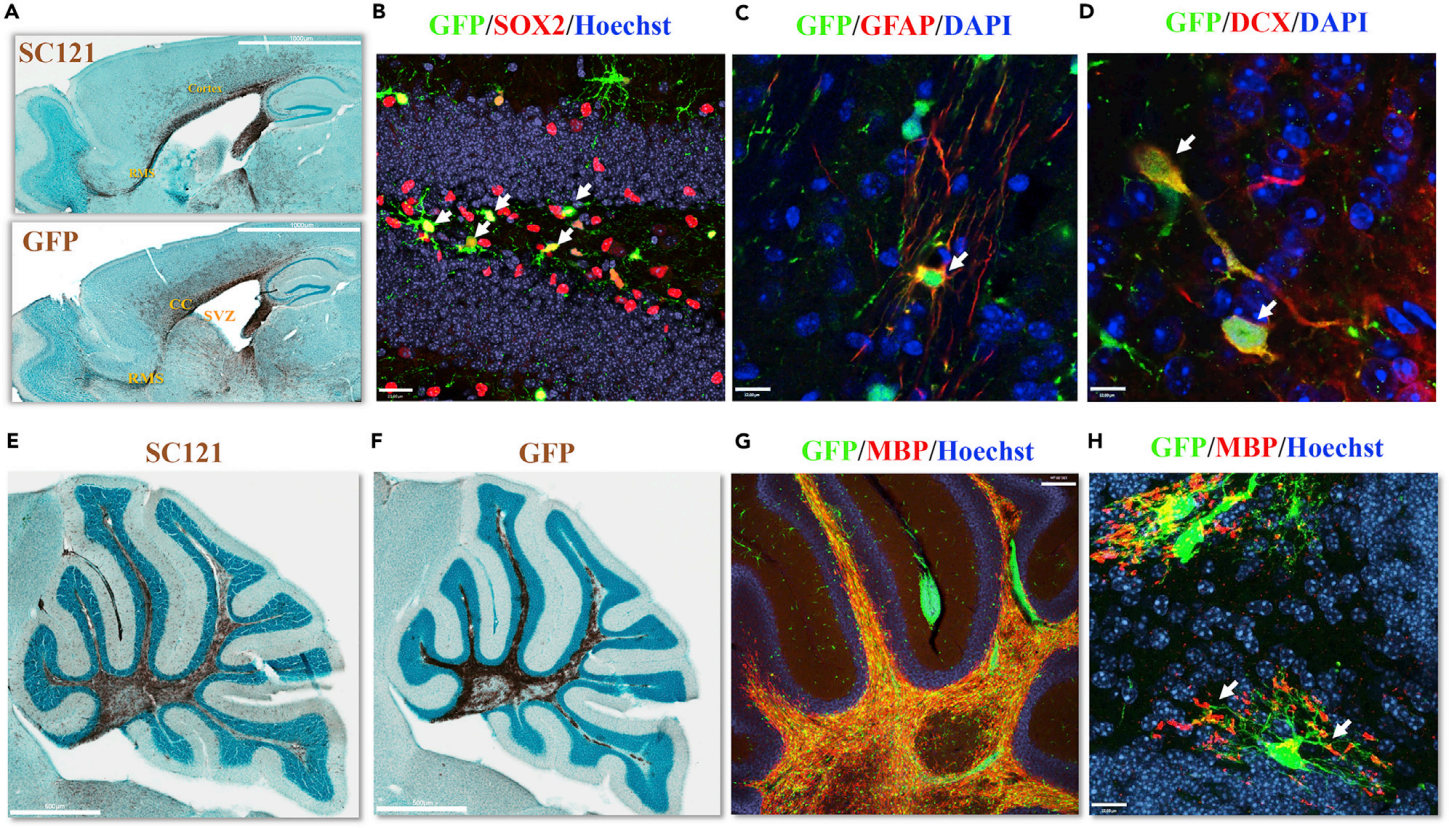
GE-NSCs Maintain Migration, NSC Markers, and Tri-lineage Differentiation Potential *In Vivo* in a Site-Appropriate Manner NSCs were targeted with a bicistronic *IL2RG* HR cassette (GFP-T2A-CD8), and then MACS-selected with CD8 microbeads, and transplanted to bilaterally target the SVZ of neonatal shi/shi-id (Figures 3A and 3F–3H) or shi/+-id (Figures 3B and 3C) mouse brains. (A) (Top) SC121 staining at 12 weeks post-transplant demonstrated extensive migration of GE-NSCs from the RMS to olfactory bulb, and white matter tracts included the corpus callosum, fimbria of the fornix, and those in the cerebellum. (Bottom) GFP staining of sibling brain sections revealed that the transplanted GE-NSCs continued to express GFP transgene in the mouse. Robust transgene expression is similar to SC121 staining. Sagittal brain sections show that the grafted cells are present in the SVZ. In addition, migrating human cells are detected in the rostral migratory stream (RMS), corpus callosum, and cortex. (B–D) Twenty four weeks post-transplantation confocal images were taken of (B) the dentate gyrus of the hippocampus stained with anti-GFP (green) and anti-Sox2 (red) Hoechst 33345 counter staining (blue) revealing that the GE-NSC migrated and maintained NSC marker, Sox 2 (arrows). Scale bar, 23 μm. (C) Anti-GFP (green), anti-human GFAP (red), and DAPI counter staining (blue) in the white tract bundle of the striatum. Scale bar, 12 μm. (D) Anti-GFP (green), anti-doublecortin (DCX) (red), and DAPI counterstaining (blue) in the olfactory bulb. Scale bar, 12 μm. Arrows highlight MACS-based enrichment of CD8 or tCD19, respectively. (E–H) GE-NSCs were transplanted directly into the cerebellum of juvenile shi/shi-id mutant mouse brains. Brain sections were processed 8 weeks post transplantation. (E) Immunostaining with human-specific SC121 monoclonal antibodies at 8 weeks post-transplant demonstrated extensive migration of GE-NSCs within white matter tracts of the cerebellum. Scale bar, 500 μm. (F) Immunoperoxidase staining of a sibling section (of Figure 3E) with anti-GFP antibody reveals a similar distribution of transgene expression as SC121 staining. Scale bar, 500 μm. (G and H) Confocal images of the white tract of cerebellum with anti-GFP (green) and anti-MBP (red) demonstrate (G) continuous GFP expression and myelin production. Injection site is indicated as shown. Scale bar, 150 μm. (H) Higher magnification from a different animal shows that progeny of gene-edited (GFP) human neural stem cells differentiate into oligodendrocytes that produce myelin basic protein (white arrows). Scale bar, 12 μm.

While we have shown that we can target NSCs for HR using the CRISPR/Cas9 system and after engineering they retained marker expression consistent with NSC function, we investigated if GE-NSCs still retain their NSC biological activity as previously shown for non-edited NSCs (Tsukamoto et al., 2013). To test this hypothesis, we targeted the *IL2RG* locus in NSCs with a GFP-T2A-CD8α cassette (Figure 1A), expanded GE-NSCs, and then transplanted them into immunodeficient mice. CD8α-purified GE-NSCs were transplanted bilaterally into the subventricular zone (SVZ) of neonatal shiverer-immunodeficient (shi/shi-id) mice, and then after 12 weeks, serial forebrain sections from the same animal were analyzed for engraftment of human modified cells by immunohistochemistry (Figure 3A). Immunoperoxidase staining with the human-specific mAb SC121 (Figure 3A top) and GFP (Figure 3A bottom) detected engraftment of GE-NSCs in the cortex and corpus callosum and importantly, showed migration of cells from the SVZ to the olfactory bulb along the rostral migratory stream (RMS) (Figure 3A). GFP expression was very similar to human-specific SC121 expression, indicating that GE-NSCs can engraft and migrate in the SVZ/olfactory system, comparable to non-edited NSCs reported previously (Tsukamoto et al., 2013).

The hallmark of human NSCs is their ability to self-renew while differentiating into neuronal, astrocytic, and oligodendrocytic lineages in long-term xenograft transplantation studies. GFP^+^ cells were detected in the hippocampus in shi/+ -id mice 24 weeks after transplant demonstrating long-term survival and migration of the GE-NSCs. In addition, SOX2-expressing human cells were detected in the subgranular layer of the dentate gyrus of the hippocampus, suggesting the maintenance of the human NSC in the neurogenic niche (Figure 3B arrows). Confocal microscopy on brain sections were performed to confirm the ability of the GE-NSC to differentiate into astrocytic and neuronal cells using the markers, glial fibrillary acidic protein (GFAP) and doublecortin (DCX), respectively. We observed GFP-positive cells costained with GFAP in the corpus callosum or white matter bundle of the striatum (Figure 3C). Furthermore, GE-NSCs migrated into the RMS and differentiated into DCX^+^ neuronal lineage in the olfactory bulb (Figure 3D), revealing that migration and differentiation of GE-NSC into astrocytic and neuronal lineages occurs as expected.

The differentiation potential of GE-NSCs into oligodendrocytes was evaluated using the oligodendrocyte mutant shi/shi-id as described previously (Uchida et al., 2012). The shi/shi-id mouse has a major deletion in myelin basic protein (MBP) gene, rendering oligodendrocytes deficient at producing myelin in the white matter tracks. Eight weeks post-transplant, GE-NSCs migrated within the white matter tract of the cerebellum (Figure 3E) and expressed the knocked in transgene GFP (Figure 3F). Immunofluorescence staining revealed that the engrafted cells in the white matter co-expressed GFP and MBP (Figure 3G). In a higher magnification of a different mouse brain, we observed that human GFP^+^ cells extended their processes and MBP expression was localized at the tip of their process, indicative of myelinating oligodendroctyes (Figure 3H). Thus, GE-NSCs maintained the potential to engraft, migrate, self-renew, and differentiate into the three CNS lineages in a site-appropriate manner as we have seen for unedited NSCs (Uchida et al., 2000, Uchida et al., 2012, Tamaki et al., 2009). In addition, the unedited NSCs have not demonstrated tumor formation in mouse or humans and the edited NSCs similarly did not generate tumors following transplantation into mice.

### GALC Lysosomal Enzyme-Overexpressing NSCs Cross-Correct Deficient Enzyme Activity in Krabbe Disease Cells

**Figure 4:**
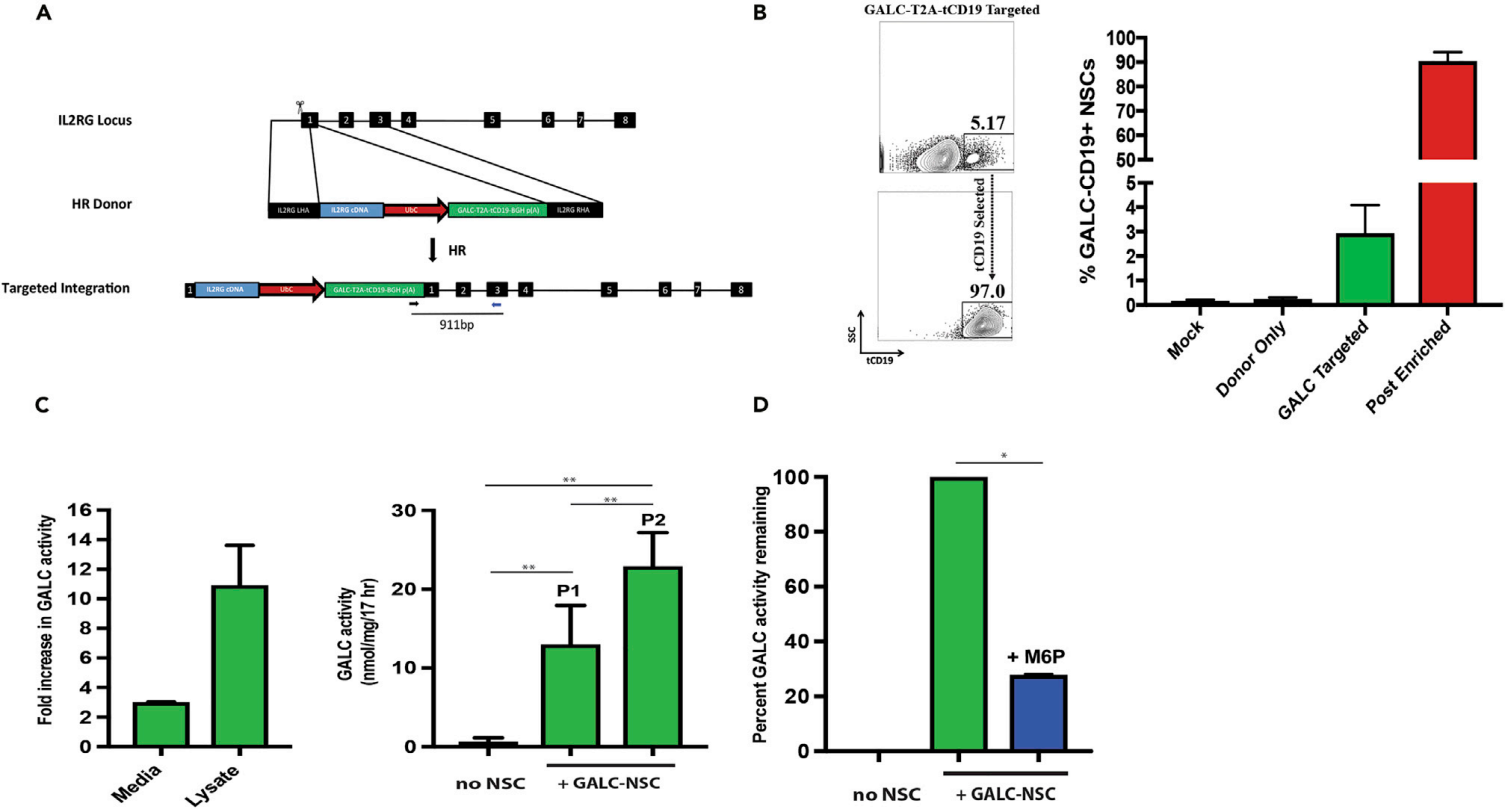
GALC Overexpressing NSCs Cross-correct Krabbe Disease Fibroblasts through Mannose-6-phosphate Receptor Pathway (A) The *IL2RG* locus was targeted for homologous recombination (HR) by creating double-strand breaks (DSBs) using Cas9 (scissors) and supplying a homologous donor template (with flanking 800-bp arms around the transgene to be inserted). Following HR, *IL2RG* cDNA is knocked in to the endogenous start codon followed by a UbC-driven cassette with GALC-T2A-tCD19 for overexpression of GalC enzyme and enrichment of targeted cells using CD19 MACS selection. (B) (Left) NSCs were nucleofected as described previously with Cas9 mRNA, MSP *L2RG* sgRNA, and the GALC-T2A-tCD19 HR donor construct. Representative fluorescence-activated cell sorting plots highlight stably integrated *GALC* construct as measured by tCD19 expression at 30 days post-nucleofection before (top) and after (bottom) enrichment of tCD19^+^ cells (right). (Right) Frequencies of GALC-NSCs before (targeted) and after MACS selection (post enriched), N = 3–6. (C) (Left) GALC enzyme assay was performed on culture media and cellular protein lysates from unedited NSCs and GALC GE-NSCs (two different NSC cell banks). GalC enzyme activity is presented as nmol/mg/17 h (normalized to unedited HuCNS-SCs). N = 1–4, two independent biological experiments. (Right) Krabbe fibroblasts (cell lines #6806 and #8304) were either cultured alone (no NSCs) or co-cultured with GALC GE-NSCs for 7 days (P1) or 14 days (P2). Krabbe fibroblasts were then harvested for GALC enzyme assay. GALC activity is presented as nmol/mg/17 h, N = 4–6, three independent biological experiments **p < 0.01, Student's t test. (D) GALC cross-correction experiments were performed as described in Figure 4C, except mannose-6-phosphate (M6P) was added to the culture media every 24–48 h (2.5 mM final concentrations) for 7 days total. Krabbe fibroblasts were harvested, protein was isolated, and GALC activity was carried out. N = 2, two independent biological experiments, *p < 0.0143, Mann-Whitney test. Data are represented as mean ± SEM.

LSDs are a group of more than 50 inherited monogenic metabolic disorders with accumulated toxic materials that primary result from the deficiency of lysosomal enzyme (Parenti et al., 2015). Among them, globoid cell leukodystrophy, or Krabbe disease, is a type of LSD that mainly manifests in the central and peripheral nervous systems due to the loss of the enzyme galactosylceramidase (*GALC*), which causes death of myelin-producing oligodendrocytes and Schwann cells, respectively. NSCs have been transplanted into the brains of patients with NCL to deliver the missing enzyme (Selden et al., 2013) and in patients with PMD to provide myelin-producing cells (Gupta et al., 2012). As we have shown that GE-NSCs produced myelin comparable to unedited NSCs, we reasoned that GE-NSCs overexpressing GALC protein would be superior in terms of producing enough GALC to enable cross-correction of damaged myelin-producing cells. Moreover, these cells could also replace already lost oligodendrocytes with “normal”-GALC expressing oligodendrocytes. Therefore we created an HR donor to knock in *GALC* (*IL2RG* cDNA-UbC-*GALC*-T2A-tCD19) into the *IL2RG* locus and also a tCD19 selection cassette to enable robust MACS-based enrichment (Figure 4A). NSCs were electroporated with the “All RNA” *IL2RG* CRISPR/Cas9 platform and the *GALC* HR donor with a mean of 2.95% CD19-positive NSCs after episomal donor DNA was diluted out at least four passages post-targeting (Figure 4B). Consistently, tCD19 MACS beads enriched to >90% of GALC GE-NSCs (Figure 4B) that accordingly maintained neuronal stem cell phenotypes (Figure S6A, related to Figure 4) and enrichment of safe harbor *IL2RG* on-target integration events (Figure S6B, Related to Figure 4).

To confirm that GALC NSCs were overexpressing functional enzyme, we performed an *in vitro* GALC enzyme assay that was validated by comparing GALC knockout Krabbe disease fibroblasts (Figure S6C, related to Figure 4) with GALC K562 cells that were generated by knocking UbC-GALC-T2A-GFP into the *IL2RG* locus (Figure S6D, related to Figure 4). Accordingly, GALC GE-NSCs expressed 10-fold more GALC enzyme than unedited NSCs in whole protein lysates and 3-fold more in the cultured media (Figure 4C, left). As GALC GE-NSCs are overexpressing active GALC enzyme and releasing it into the culture media, we performed co-culture experiments with previously characterized Krabbe disease fibroblasts (Luzi et al., 1995, Rafi et al., 1995) to ascertain if they could be cross-corrected for GALC enzyme activity by the GALC NSCs. After 7 days (1 passage) and 14 days (2 passages) of trans-well-mediated co-culturing of GALC NSCs with Krabbe disease fibroblasts, GALC enzyme activity was increased 18- and 34-folds, respectively (Figure 4C, right). These results suggest that active GALC enzyme is accumulating linearly as the co-culture time increases. To confirm that GALC cross-correction was mediated through the essential lysosomal mannose-6-phopshate (M6P) receptor pathway (Tamaki et al., 2009), we cultured with M6P to antagonize the enzyme uptake in Krabbe disease fibroblasts. We were able to inhibit 73% of GALC enzyme uptake with M6P added to the culture media every 24–48 h (Figure 4D), suggesting that the cross-correction of GALC enzyme activity to Krabbe disease cells is via the M6P receptor lysosomal pathway.

We also confirmed that *GALC* GE-NSCs retained their NSC biological characteristics by transplanting *GALC* GE-NSCs into the cerebellum of juvenile Shi-id homozygous mice. Immunohistochemical analyses showed engraftment via SC121 marker expression and myelin expression via MBP expression of *GALC* GE-NSCs (Figure S7, related to Figure 4). These data combined with the fact that GALC-NSCs maintain neuronal stem cell markers (Figure S6A, related to Figure 4) highly suggest that *GALC* GE-NSCs retain their NSC biological characteristics and may be of therapeutic potential, which warrants future safety and efficacy investigation in relevant mouse models for the treatment of Krabbe disease and other demyelinating CNS disorders.

## Discussion

We have outlined a genome editing methodology in NSCs using the CRISPR/Cas9 platform. In brief, we demonstrate (1) genome editing by HR at multiple NSC safe harbor loci; (2) an enrichment strategy for selecting >90% of genome-edited NSCs (GE-NSCs) by using signaling-incompetent cell surface markers and microbead technologies; (3) that GE-NSCs retain their NSC characteristics *in vivo* by showing proliferation in the hippocampal stem cell niche, migration along the rostral migratory stream, and differentiation into neurons and astrocytes as well as oligodendrocytes that produce MBP in all brain regions investigated; and (4) generation of GE-NSCs overexpressing the GALC enzyme that cross-correct GALC enzyme activity in Krabbe disease cells, and engraft and produce myelin *in vivo*. Overall, these data warrant more comprehensive safety and efficacy studies for the investigational GE-NSCs cell products that could be used as a therapeutic cell product for CNS disorders and also could be leveraged to study human NSC biology.

Most CRISPR/Cas9 applications in neuroscience have used NHEJ-based editing of (1) mouse brains *in vivo* and (2) human iPSCs and ESCs *in vitro*, and although these advances have helped better understand neuronal networks, there are limitations of these systems, such as nuclease delivery and restricting editing to a specific cell type (Heidenreich and Zhang, 2016). Although lentiviral-mediated gene transfer for NSCs has been shown to efficiently deliver transgenes *in vitro* and *in vivo* (Jandial et al., 2008), the concerns of insertional mutagenesis always remain high. An alternative approach is to precisely edit the locus of interest by homologous recombination directly in human NSCs that possess *in* vitro and *in vivo* multilineage potential that can be expanded and created into cell banks to provide an allogeneic product for transplantation. Because NSCs maintain trilineage potential (oligodendrocytes, neurons, astrocytes) *in vitro* and CNS site-specific differentiation and migration *in vivo*, NSCs are an ideal candidate cell population to genetically engineer to explore NSC gene function programs, as banked NSCs migrate globally to facilitate neuroprotection by supplying missing lysosomal enzyme or providing neurotrophic factors. Our data show that it is possible to target NSCs for HR at multiple loci and enrich and expand genome-edited NSCs, and more importantly, our data unequivocally demonstrate that GE-NSCs maintain hallmark NSC functions: stem cell marker expression, lineage commitment, and migration *in vivo*. Therefore, our methodology sets the framework to allow investigators to interrogate CNS gene-cell functions at any locus of interest and also to lineage trace NSCs *in vivo*.

Banked NSCs have already been tested in preclinical models as well as have been used as an investigational cellular product in multiple phase I or II clinical trials for difficult-to-treat CNS disorders, such as retinal degeneration (McGill et al., 2012, Cuenca et al., 2013), spinal cord injuries (Cummings et al., 2005, Salazar et al., 2010), rare myelin disorders (Uchida et al., 2012), and LSDs (Tamaki et al., 2009). Although the outcomes of these trials were encouraging, they also highlighted the potential to facilitate improvements to the cellular product (Tsukamoto et al., 2013). Krabbe disease is an LSD that results from the loss of GALC activity in myelin-producing cells, and as NSCs already promote oligogenesis (Uchida et al., 2012), we reasoned that constitutively overexpressing GALC through HR in NSC safe harbor loci would improve their therapeutic potential. Accordingly, our data highlight that GALC-NSCs secrete enough active GALC enzyme to be taken up by Krabbe disease cells through the M6P receptor pathway and rescue deficiencies in enzyme levels. Furthermore, our current studies also show that GALC GE-NSCs expresses essential neuronal stem cell markers and are able to differentiate into oligodendrocytes (and produce myelin) *in vivo*. The fact that GALC-NSCs can not only secrete enough enzyme but also can differentiate into oligodendrocytes is critical for their therapeutic activity. Now, the next logical set of future studies would be to investigate whether GALC GE-NSCs that overexpress GALC enzyme *in vivo* have the ability to cross-correct dysfunctional host oligodendrocytes in a Krabbe mouse model and also provide donor-derived oligodendrocytes to repair hypomyelination. In terms of future experiments, our methodology also allows us to overexpress any protein of interest, and future studies will interrogate whether overexpression of neurotrophic factors, such as brain-derived neurotrophic factor, can improve neuronal survival in neurodegenerative diseases like Alzheimer, Parkinson, and Huntington models.

In conclusion, we have set the framework for CRISPR/Cas9 genome editing by HR in human-brain-derived multipotent NSCs that will advance the field of neuroscience and treatment of CNS disorders.

### Limitations of the Study

We demonstrate that CRISPR-Cas9 gene-targeted human NSCs can engraft, migrate, and differentiate into CNS cells in a site-specific manner in the mouse brain. We also show that GALC-expressing NSCs can be engineered to secrete lysosomal enzymes that are able to cross-correct Krabbe fibroblasts *in vitro*. The limitation of this study is that we have not definitively shown *in vivo* correction of a Krabbe disease mouse model, such as the twitcher mouse model. Additional experiments are needed to further provide preclinical experimental proof of the feasibility and efficacy of using GE-NSCs as a treatment for LSDs.

## Methods

All methods can be found in the accompanying Transparent Methods supplemental file.

## Supplemental Information

Document S1. Transparent Methods and Figures S1–S7
