# Supplementary material for: CRISPR/Cas9 Genome Engineering in Engraftable Human Brain-Derived Neural Stem Cells
Source: iScience. 2019 May 4;15:524–35. doi: 10.1016/j.isci.2019.04.036 (PMC6538928; doi:10.1016/j.isci.2019.04.036)
Supplement: Document S1. Transparent Methods and Figures S1–S7 [file mmc1.pdf]

ISCI, Volume *15*

Supplemental Information

CRISPR/Cas9 Genome Engineering in Engraftable Human Brain-Derived Neural Stem Cells

Daniel P. Dever, Samantha G. Scharenberg, Joab Camarena, Eric J. Kildebeck, Joseph T. Clark, Renata M. Martin, Rasmus O. Bak, Yuming Tang, Monika Dohse, Johannes A. Birgmeier, Karthik A. Jagadeesh, Gill Bejerano, Ann Tsukamoto, Natalia Gomez-Ospina, Nobuko Uchida, and Matthew H. Porteus

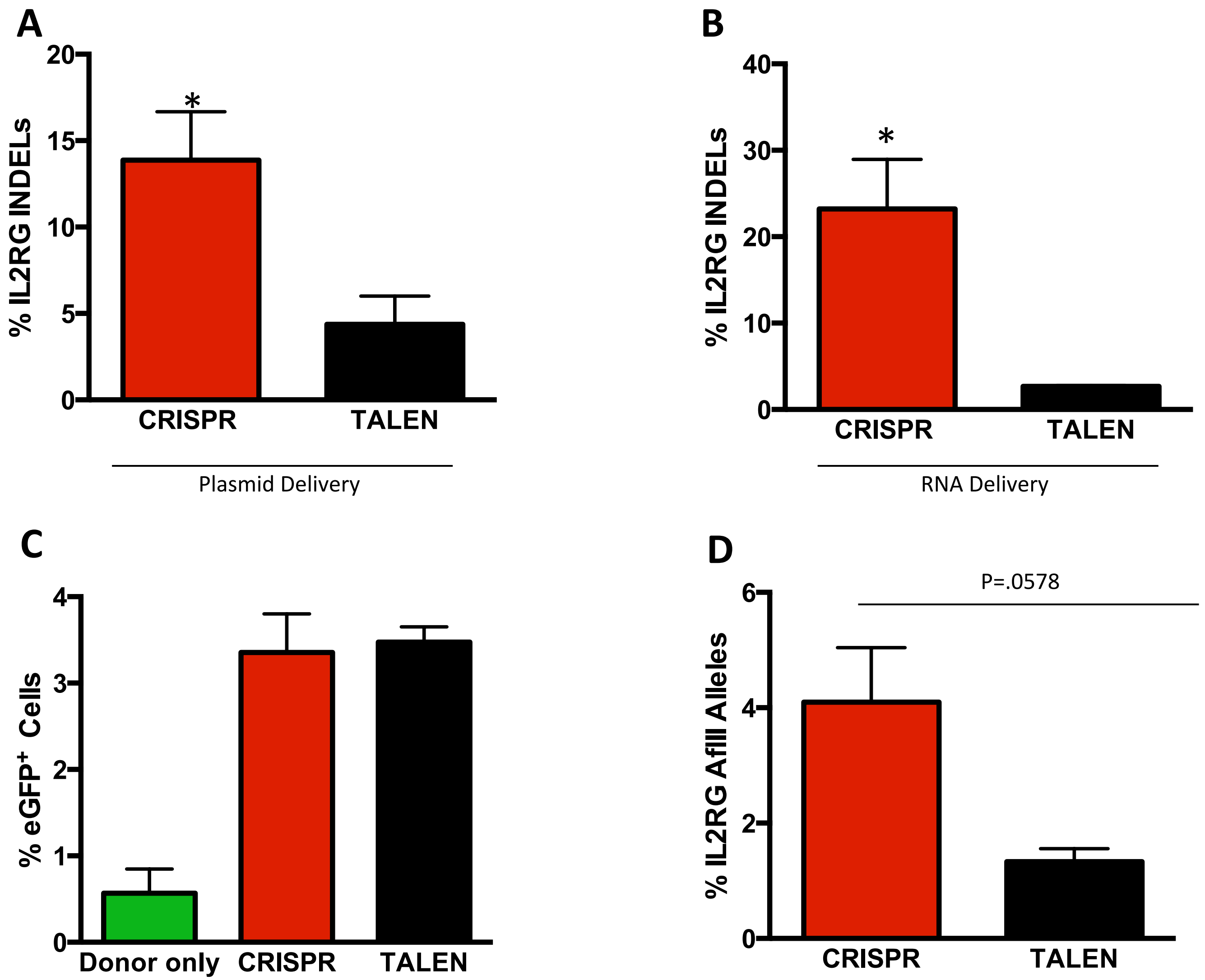

Supplemental Figure 1. The CRISPR system is highly active in human NSCs. A) 500,000 NSCs were electroporated (plasmid delivery) with either the CRISPR/Cas9 system or TALEN pairs that were designed to recognize the human *IL2RG* locus. Seven days post targeting, gDNA was harvested, *IL2RG* alleles were amplified by PCR with primers that overlapped the cut site, and TIDE was run to analyze INDEL frequencies. (N= 4-7), * $p < 0.05$, Student's T-test. **B)** NSCs were electroporated as described above with Cas9 mRNA and MSP sgRNA or TALEN pairs delivered as mRNA, and then *IL2RG* alleles were analyzed by TIDE software for INDELs. (N= 2-5), * $p < 0.05$, Student's T-test. **C)** NSCs were electroporated with *IL2RG* engineered nucleases along with UbC-GFP donor templates with *IL2RG* homology arms. 30 days post-targeting, cells were harvested for FACS GFP analyses. (N= 4-6). **D)** NSCs were electroporated with *IL2RG* engineered nucleases along with a *IL2RG* homologous donor template intended to introduce a AflII restriction site following homologous recombination. 7 days post-targeting, gDNA was harvested, then *IL2RG* alleles were amplified to produce a 1.6kb product. Amplified alleles were digested with AflII and run a PAGE gel. The number of HR alleles were calculated as follows: ((digested alleles/ digested alleles) + undigested alleles). (N= 4-6), Student's T-test. Data are represented as mean +/- SEM.

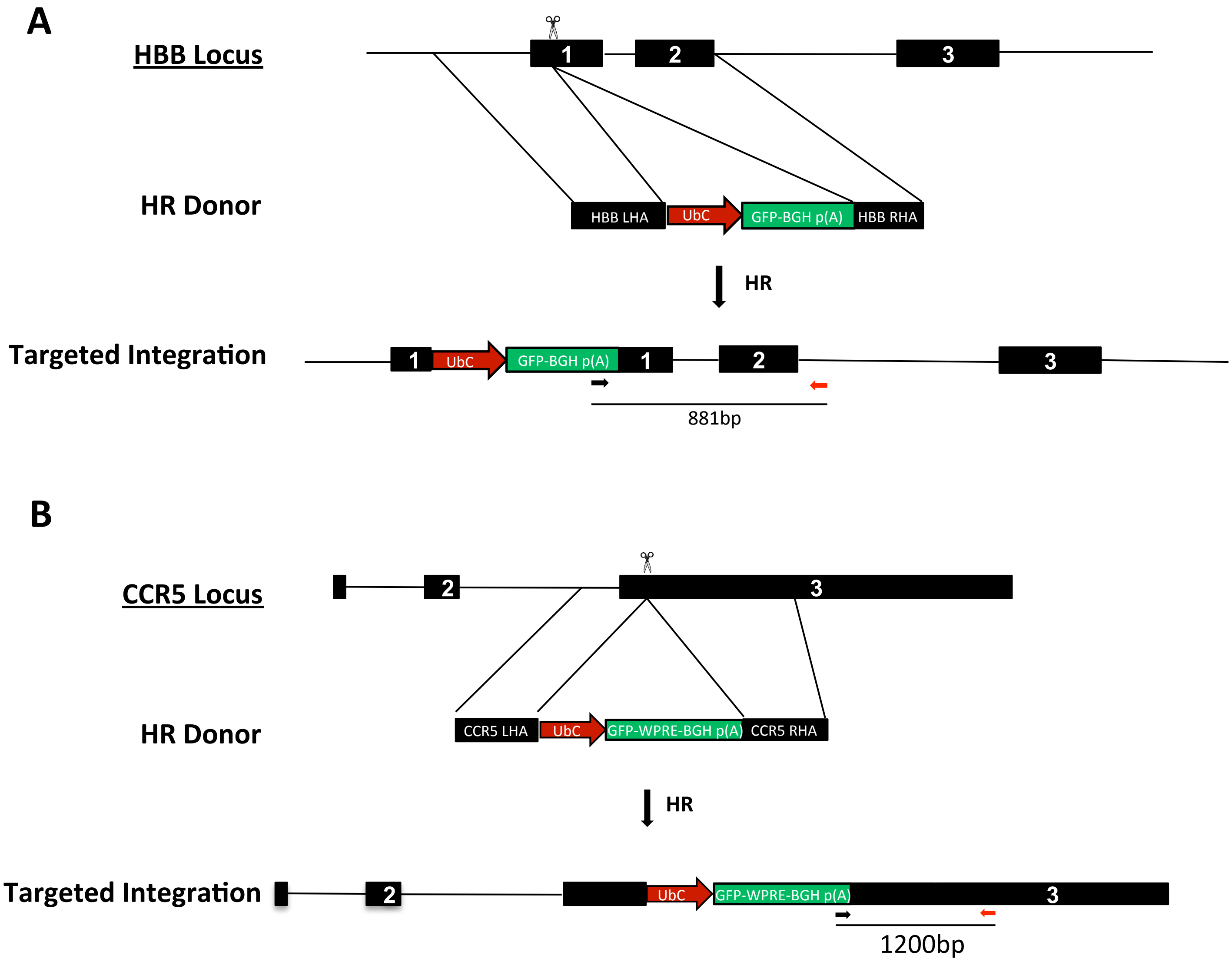

Supplemental Figure 2. Schematic of targeted integration into *HBB* and *CCR5* loci. A) The HBB locus was targeted by creating a DSB in exon 1 via Cas9 (scissors) and supplying a UbC-GFP homologous donor template. Alleles with integrations were identified by PCR (881bp) using an In **(black)** - Out (**red**) primer set. **B)** The *CCR5* locus was targeted in exon 3 as described above. PCR identified integrated (1200bp) alleles using In **(black)** - Out (**red**). Loading control for all In-Out PCRs was the wildtype *CCR5* allele

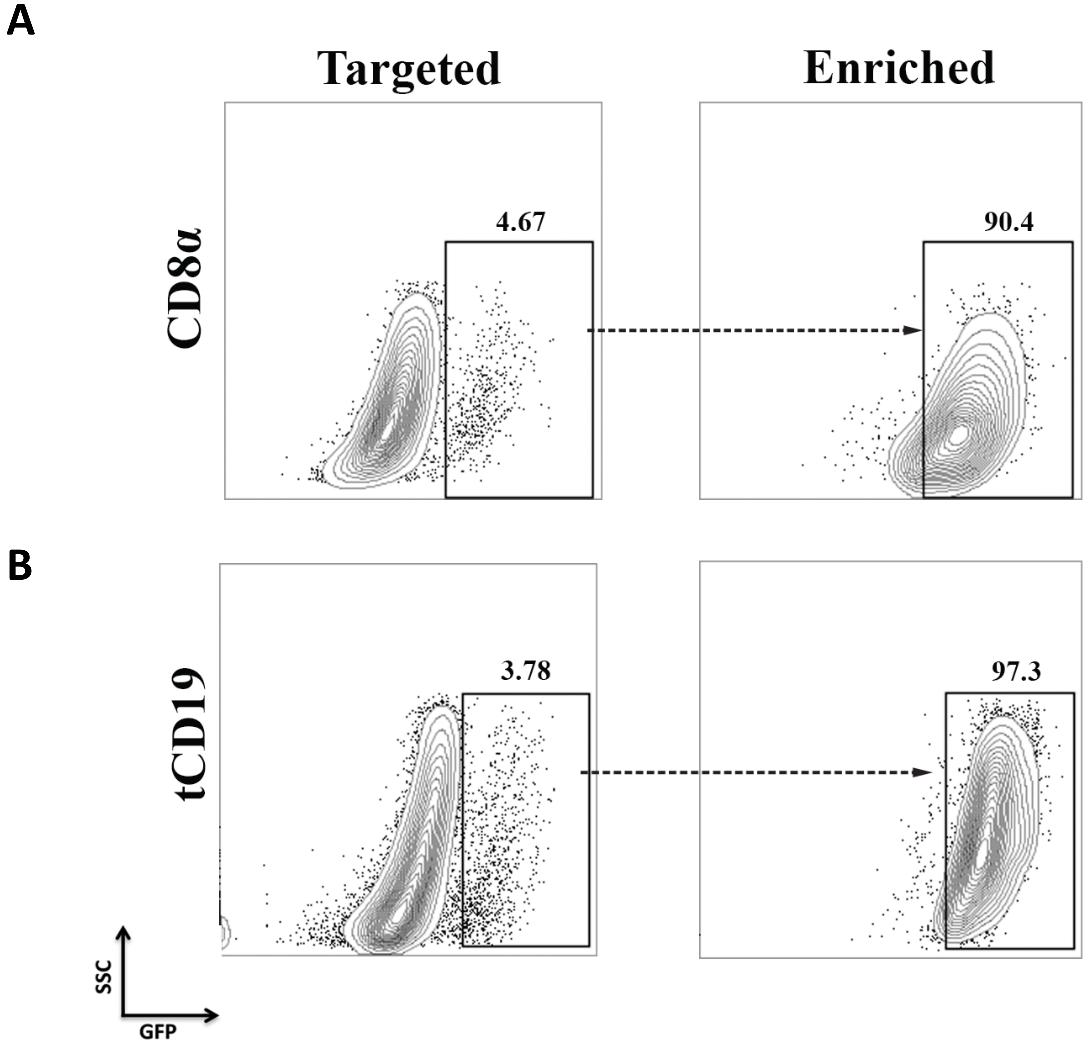

Supplemental Figure 3. Representative FACS images showing MACS enrichment of IL2RG targeted NSCs. A bicistronic *IL2RG* HR cassette was created that separate GFP from CD8α or truncated CD19 (tCD19) via a T2A peptide motif to allow robust magnetic bead enrichment of *IL2RG*-targeted NSCs. NSCs were nucleofected with 2ug HR donor and 1ug plasmid encoding Cas9 and sgRNA. Cells were grown for 30 days to allow episomal HR donor to dilute out during proliferation of NSCs. **A)** Representative FACS plots show a population of NSCs with stable integration of the bicistronic GFP-T2A-CD8 cassette before enrichment at day 30 after electroporation (left), and 30 days after enrichment using Magnetic Activated Cell Sorting (MACS) CD8 Microbead technologies (right). **B)** Representative FACS plots showing a population of NSCs with stable integration of the bicistronic GFP-T2A-tCD19 cassette before enrichment at day 30 after electroporation (left), and a population enriched of genome-modified NSCs using MACS CD19 Microbead technologies (right)

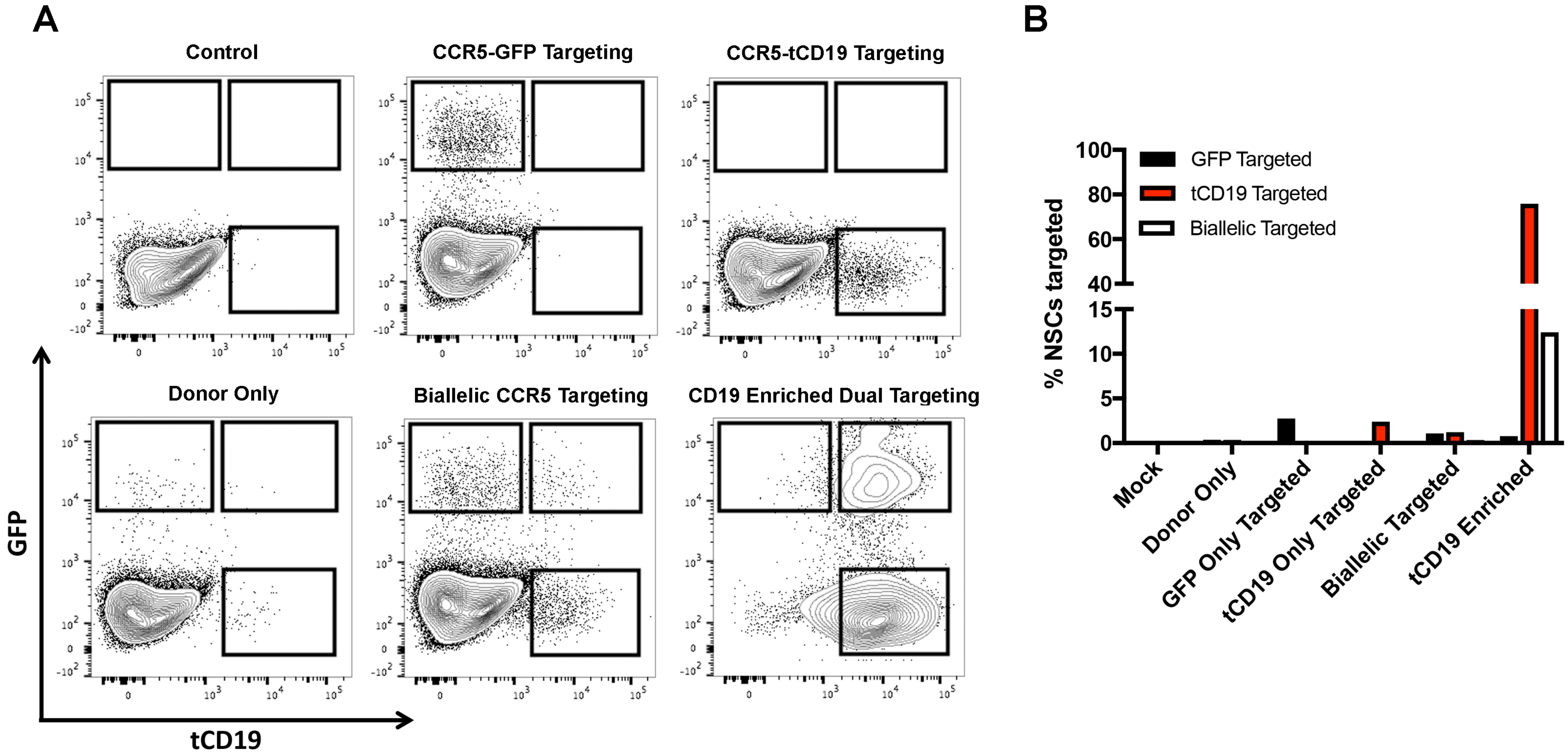

Supplemental Figure 4. Enrichment of biallelic CCR5-targeted NSCs. **A)** Human NSCs were targeted at *CCR5* with a UbC-GFP cassette (top middle), with a UbC-mCherry-T2A-tCD19 cassette (top right), or with both constructs (bottom middle). CD19-based MACS enrichment results in greater than a 10 fold increase in biallelically edited *CCR5* NSCs as well as monoallelic *CCR5* edited cells. **B)** Quantification of the percent of GFP targeted, tCD19 targeted or biallelically targeted NSCs from the experiment shown in **A**.

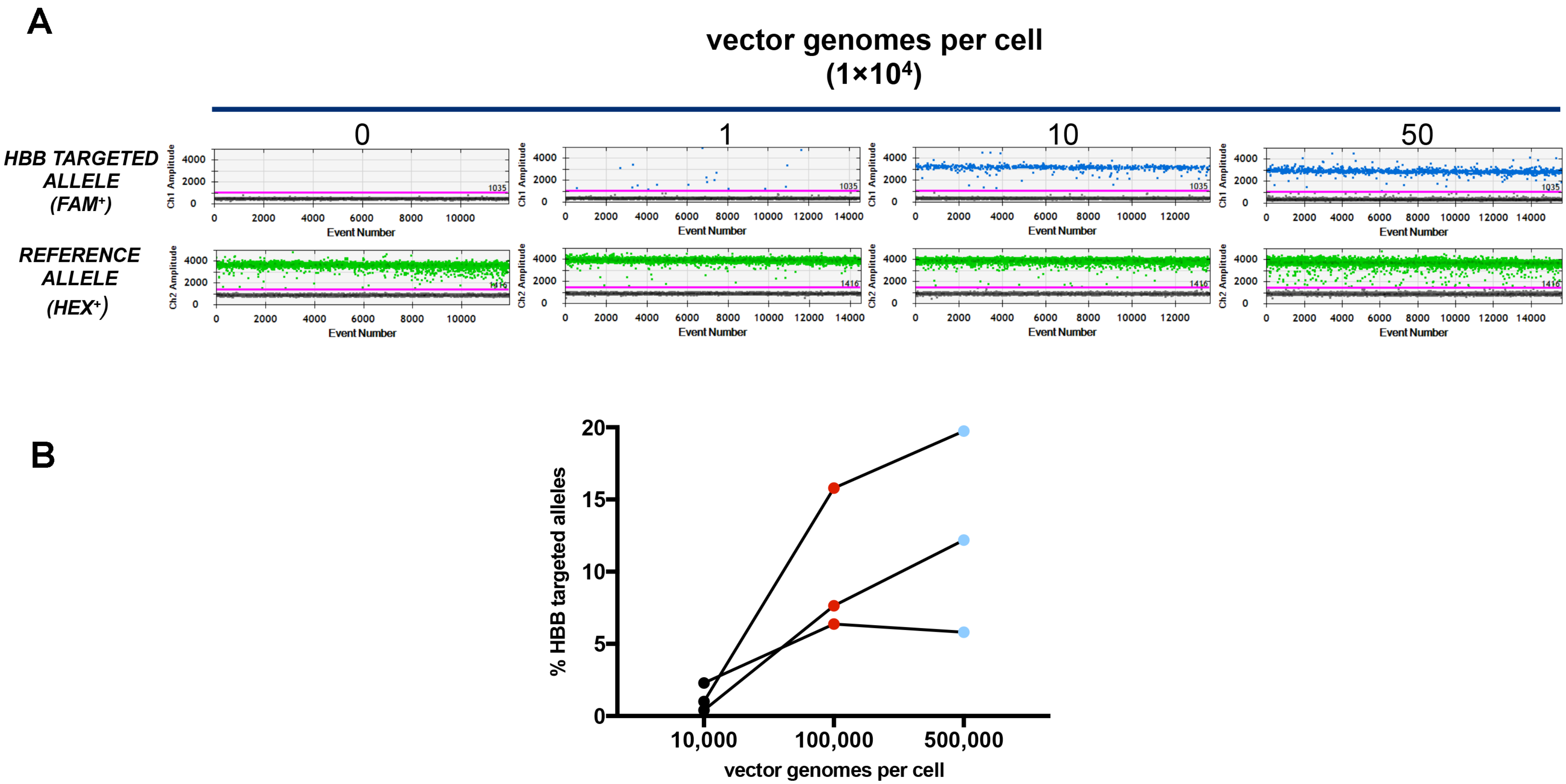

Supplemental Figure 5: Recombinant adeno-associated virus serotype six (AAV6) is a suitable homologous donor template in human NSCs. 500,000 human NSCs were electroporated with Cas9 mRNA and HBB-specific MSP sgRNAs and then cells were immediately transduced with rAAV6 carrying UbC-GFP with arms of homology for HBB (centered on the sgRNA cut site). **A)** Representative ddPCR images show the increased number of HBB targeted allele positive droplets when the number of AAV6 vector genomes per cell is increased. The aggregate data is shown in **B** below. The reference allele amplified is *CCR2*. **B)** In three independent experiments, NSCs were *HBB* targeted with a UbC-GFP AAV6 donor (at three different VGs/cell), cells were harvested 7 days later and then gDNA was harvested and ddPCR was carried out to determine the frequency of *HBB* integrated alleles. Using AAV6 VGs/cell of 10,000, 100,000 and 500,000, mean integration allele frequencies were 1.26%, 9.93%, and 12.57%, respectively. The lines pair VG dose responses in the same experiment.

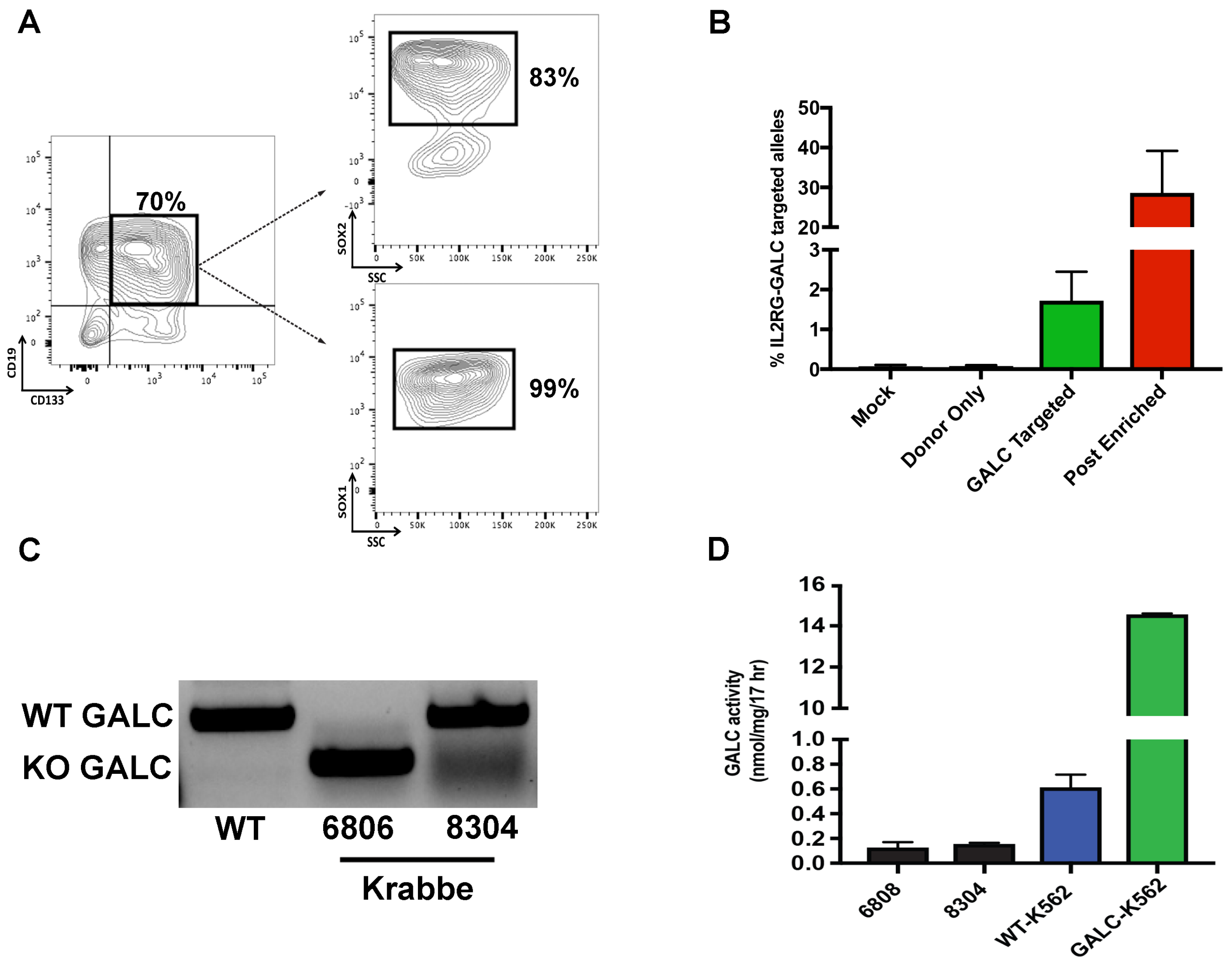

Supplemental Figure 6: GALC-NSCs maintain neuronal stem cell markers and establishment of GALC enzyme assay. **A)** Enriched GALC-NSCs were analyzed for editing via tCD19 and stem cell markers via CD133, SOX2 and SOX1. 70% of GALC-NSCs were targeted and express quintessential neuronal stem cells markers. **B)** 500,000 NSCs were mock electroporated, electroporated with DNA donor only, or electroporated with GALC-T2A-tCD19 DNA donor and ALL RNA Il2RG CRISPR components. After 30 days in culture, Mock, Donor Only, or half of the GALC targeted cells were harvested for gDNA, while the other half od GALC targeted cells were CD19 enriched and expanded for another 7 days before gDNA harvest. N=3-5. **C)** gDNA from WT fibroblasts or Krabbe Disease fibroblasts from two patients (purchased from Coriell; 6806 and 8304) were analyzed for GALC deletion as described in the materials and methods. Cell line 6806 is a homozygous knockout for the deletion and 8304 is heterozygous for the GALC deletion. **D)** GALC-K562 cells were generated to establish the GALC enzyme activity assay. These data show that the 6806 and 8304 Krabbe fibroblast cell lines are deficient in GALC enzyme activity. Data are represented as mean +/- SEM.

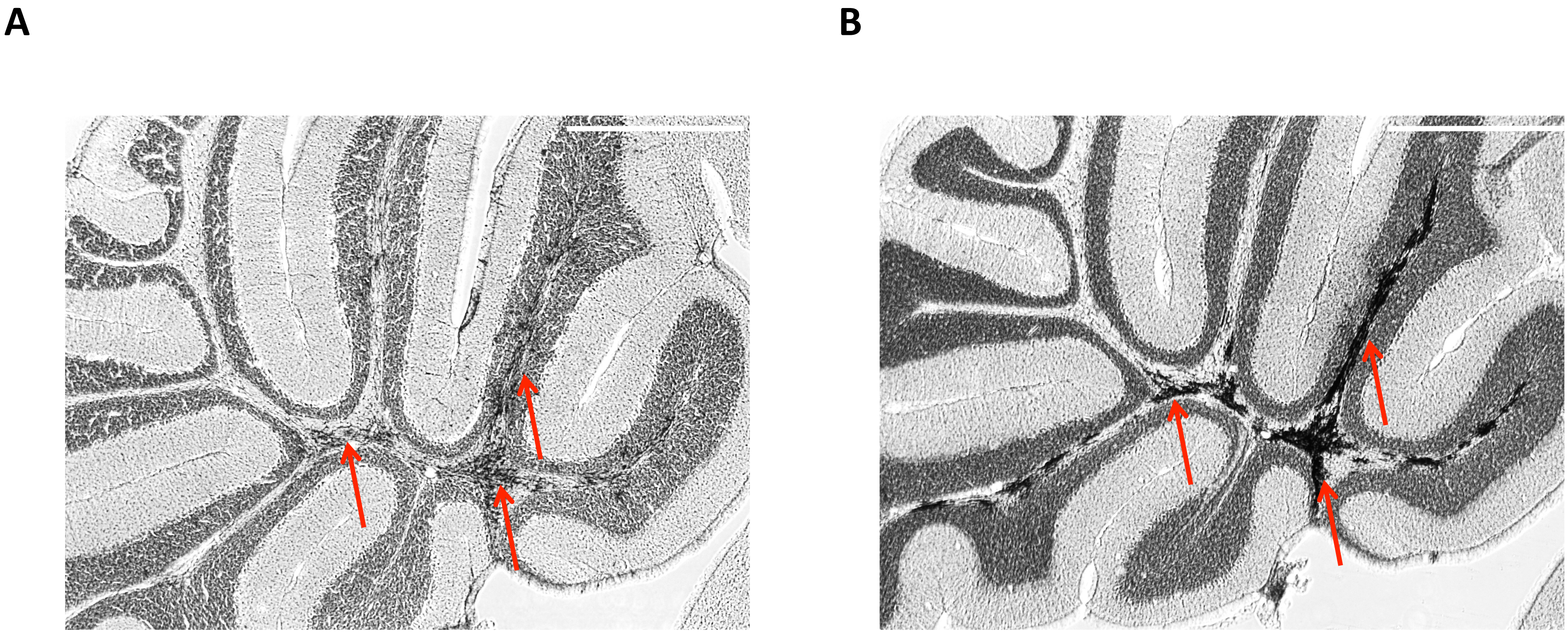

Supplemental Figure 7: GALC-NSCs engraft in the mouse cerebellum and produce myelin *in vivo*. A) immunoperoxidase staining with the human-specific mAb SC121 (brown with red arrow) detects engraftment of human cells in the white matter in the cerebellum. **B)** immunoperoxidase staining of a sibling section with anti-MBP (brown with red arrows) reveals a similar distribution of grafted GE-NSCs. Scale bars shown are 500µm. These data show that human cells that engrafted after 8 weeks are producing myelin.

Transparent Methods:

NSC cell culture

Human NSCs grown as neurospheres were generated as described under non-GMP conditions(Uchida et al., 2000, Uchida et al., 2012). were cultured at a density of 1E5 per ml in X-VIVO 15 medium (Lonza) supplemented with N2 (Invitrogen, 1:100), heparin (2 ug/ml(, N-acetylcysteine (63ug/ml), fibroblast growth factor 2 (20 ng/ml), epidermal growth factor (20 ng/ml), and leukemia inhibitory factor (10 ng/ml). Neurospheres with Passage 8-13 were used in this study. Nueropsheres were disoociated by purified collagenase (Liberage/Blenzyme, Rohche) into single cell suspension and replated for passage in the medium described above.

Genome Editing Reagents:

TALEN-mediated genome editing: IL2RG TALENs were synthesized (GenScript) using the Δ152 N-terminal domain and the +63 C-terminal domain and fused to the FokI nuclease domain and cloned into pcDNA3.1 (Invitrogen) as described(Hendel et al., 2014). CRISPR/Cas9-mediated genome editing: sgRNA expression vectors were constructed by cloning of 20 bp oligonucleotide target sequences into px330-U6-Chimeric_BB-CBh-hSpCas9 (a kind gift from Feng Zhang, Addgene plasmid #42230) containing a human codon-optimized SpCas9 expression cassette and a human U6 promoter driving the expression of the chimeric sgRNA(Hendel et al., 2014). The genomic target sequences for the guides were as follows: HBB: 5′-CTTGCCCCACAGGGCAGTAA-3′, CCR5: 5′ GCAGCATAGTGAGCCCAGAA-3′, IL2RG: 5′ -GGTAATGATGGCTTCAACA-3′. Chemically modified sgRNAs: Cas9 was delivered as 5meC, Ψ-modified mRNA (TriLink BioTechnologies) and sgRNAs were synthesized by Agilent Technologies (MS and MSP), Synthego (MS) or TriLink BioTechnologies (MS) with 2′-*O*-methyl (M), 2′-*O*-methyl 3′phosphorothioate (MS), or 2′-*O*-methyl 3′thioPACE (MSP) incorporated at three terminal nucleotides at both the 5′ and 3′ ends, and thus represents the “All RNA” delivery of the CRISPR system(Hendel et al., 2015). Targeting vectors: *CCR5* and *IL2RG* plasmid targeting vectors had ~2 × 800 bp arms of homology, which were generated by PCR amplification of the corresponding

loci using genomic DNA isolated from K562 cells. *HBB* had homology arms of 540bp and 420bp. The homology arms were cloned into a ~2,900 base pair vector based on pBluescript SK^+ using standard cloning methods(Hendel et al., 2015, Hendel et al., 2014). Between the homology arms, donors contain the Ubiquitin C promoter (UbC) driving expression of GFP (for both HBB plasmid and AAV6 donors). For CCR5 biallelic editing, one plasmid construct had UbC-GFP and the other had UbC-mCherry-T2A-tCD19. The *IL2RG* homologous donor construct had an *IL2RG* cDNA upstream of the UbC promoter and GFP (See Figure 1A). Alternatively, bicistronic cassette constructs separating proteins by a self-cleaving 2A peptide were generated as follows: GFP-2A-CD8 (CD8 alpha cell surface marker for purification of GE-NSCs), GFP-2A-tCD19 (truncated cell surface marker for purification of GE-NSCs) and GALC-2A-tCD19 (therapeutic enzyme construct for Krabbe disease with tCD19).

Genome Editing of NSCs

Single suspension of 500,000 NSCs were transfected with 1 µg TALEN-encoding plasmids and 1-2 µg donor plasmid (unless otherwise indicated) by nucleofection (Lonza) with an Amaxa 4D Nucleofector (program CA137) with the P3 Primary Cell Nucleofector Kit (V4XP-3032) using 20 µL, 16-well Nucleocuvette strips following manufacturer's instructions. Alternatively, 2.5 x 10^6 NSCs were transfected with 5 µg TALEN-encoding plasmids and 5-10 µg donor plasmid (unless otherwise indicated) by nucleofection (Lonza) with an Amaxa 4D Nucleofector (program CA137) with the P3 Primary Cell Nucleofector Kit (V4XP-3024) using 100 µL Nucleocuvettes following manufacturer's instructions. For experiments with chemically modified sgRNAs and Cas9 mRNA, 500,000 NSCs were electroporated with 15µg Cas9 mRNA and 10µg MS or MSP sgRNA. After nucleofection, NSCs were plated in culturing flasks and cultured for multiple passages.

Genome Editing of NSCs with Cas9 mRNA and AAV6 donors

A single cell suspension of 500,000 NSCs were responded with 15µg of Cas9 mRNA was mixed with 10µg of sgRNA in 20 uL of P3 solution (Lonza). Nucleofection was performed using 16-well Nucleocuvette Strip with the 4D Nucleofector system (Lonza)

using CA137 code. Immediately after electroporation, cells were transferred into one well of a 48 well plate containing 250 µl of NSC media. Then the HBB UbC-GFP AAV6 donor vector (purchased from Vigene Biosciences) was added directly to the electroporated cells at vector genomes/cell of 10,000, 100,000, or 500,000. After 24 hours, cells were transferred into a T25 flask with 5ml of NSC media and cells were harvested and gDNA was extracted 6 days later (7 days total post electroporation).

Analysis of homologous recombination (HR) via flow cytometry and 'In-Out' PCR

To monitor homologous recombination (HR), GE-NSCs were analyzed for GFP expression at each passage after nucleofection. GFP expression was measured on an Accuri C6 flow cytometer (BD Biosciences, San Jose, CA, USA). Alternatively, expression of transgene CD8 or CD19 was assessed by flow cytometry at each passage. At passage, neurospheres were dissociated as described above and single cell suspension were immunostained with anti-CD8-APC or anti-CD19-APC (Miltneyi Biotech) following manufacture's instructions. 'In-Out' PCR was performed to qualitatively identify integration at the intended genomic locus where one primer binds outside the homology arms and the other primer binds inside the donor cassette; thus amplification should only occur if the donor cassette is integrated correctly.

Digital Droplet PCR (ddPCR) analysis of on-target integration of HR donors

ddPCR was carried out to determine the frequency of allelic targeted integration in human NSCs as previously described(Vakulskas et al., 2018). Briefly, genomic DNA was extracted using QuickExtract DNA Extraction solution (Epicenter) and then digested in either HINDIII-HF (for analyzing 3' integrations into HBB) or BamH1-HF (for analyzing 3' integrations into IL2RG or CCR5) for 2 hours. 3µl of digested gDNA was then used as the template for the ddPCR reaction. Droplet samples were prepared according to manufactures protocol (Bio-Rad) and PCR cyclin conditions were used as follows: 98°C (10 min); 94°C (0.5 min); 60°C (0.5 min); 72°C (2 min; 50 cycles); and 98°C (10 min). Finally, droplets were analyzed according to the manufacturer's instructions using the QX200 system (Bio-Rad). The gene-specific integrations primer/probes were designed to analyze BGH PolyA signal at the 3' end of the

recombinant alleles. The primers used to amplify the gene-specific targeted alleles and reference alleles were as follows: HBB forward: 5’-GGGAAGACAATAGCAGGCAT-3’, HBB reverse: 5’-CGATCCTGAGACTTCCACAC-3’, HBB probe: 5’-FAM-TGGGGATGCGGTGGGCTCTATGGC-BHQ-3’, CCR5 forward: 5’-GGGAAGACAATAGCAGGCAT-3’, CCR5 reverse: 5’-TCAAGAATCAGCAATTCTCTGAGGC-3’, CCR5 probe: 5’- FAM-TGGGGATGCGGTGGGCTCTATGGC-BHQ-3’, IL2RG forward: 5’-GGGAAGACAATAGCAGGCAT-3’, IL2RG reverse: 5’-CAGATATCCAGAGCCTAGCCTCATC-3’, IL2RG probe: 5’- FAM-TGGGGATGCGGTGGGCTCTATGGC-BHQ-3’, CCRL2 (reference) forward: 5′ -GCTGTATGAATCCAGGTCC-3′, CCRL2 reverse: 5′ -CCTCCTGGCTGAGAAAAG-3′, CCRL2 probe 5′-HEX-TGTTTCCTCCAGGATAAGGCAGCTGT-BHQ-3′.

INDEL analyses using tracking INDELs by decompression (TIDE) software

Genomic DNA (gDNA) was extracted at least seven days following electroporation of engineered nucleases in NSCs. Gene-specific primers were used to amplify alleles of interest (*CCR5, HBB, IL2RG*) and purified PCR fragments were then Sanger-sequenced as described(Dever et al., 2016). INDEL frequencies were quantified using the TIDE software (Tracking of Indels by Decomposition)(Brinkman et al., 2014).

Restriction Fragment Length Polymorphism (RFLP) Allele Analysis

The introduction of an AflII restriction site between 800bp homology arms allowed analysis of HR frequencies by Restriction Fragment Length Polymorphism (RFLP) at the *IL2RG* locus. Cas9 (under CMV promoter) + sgRNA (under U6 promoter) was delivered in the px330 plasmid construct (1ug). TALEN pairs were constructed as described above using the golden gate cloning system and delivered as plasmid constructs (0.5ug of each). 500,000 NSCs were nucleofected with either 1ug HR donor or 1ug HR donor and a nuclease. Cells were allowed to grow in culture for 7 days, gDNA was harvested, In-Out PCR was performed, the PCR product was isolated, and finally digested with AflII overnight and products were run on 10% PAGE gels and visualized and quantified for band intensities via ImageJ software. The amount of modified *IL2RG* alleles was

quantified by dividing the density of cut alleles (800bp) by total alleles (unmodified (1.6kb) and modified).

Purification of GE-NSCs by Magnetic Activated Cell Sorting (MACS)

To Purify GE-NSC using either CD8 or CD19 transgene cell surface marker, either human CD8 Microbeads or CD19 MicroBeads (Milenyi Biotec) was used with following the manufacturer's instructions. Briefly, NSCs were expanded 4-6 passages after transfecting plasmids containing, Cas9, sgRNA and targeting vectors. Cells were harvested and dissociated to single cell suspensions by blenzyme and cell number was determined. Cells were centrifuged, resuspended into MACS buffer and added either CD8 or CD19 Microbeads according to manufacturer's protocol. Magnetic separation was performed by using MS column to collect magnetically labeled cells. The eluted cells were applied to a second MS column to increase the purity of CD8+ or CD19+ cells. The purity of MACS selected cells were examined by staining either antibodies against CD8-APC or CD19-APC (Miltenyi Biotec). The purified cells were for further expansded and analyzed at each passage.

Transplantation of GE-NSC

Shi mice (C3Fe.SWV-Mbpshi/J) were backcrossed with immunodeficient Rag2/IL-2Rγ knockout mice (Shi-id). For transplantation, a suspension of GE-NSCs (1E5 cells in 1 microL per site) was prepared and transplanted bilaterally into the corpus collusum, SVZ and cerebellar white matter of neonatal or juvenile Shi-id mice as described previously(Uchida et al., 2012). A total of 114 mouse brains (80 females, 34 males) were analyzed for human cell engraftment by human specific monoclonal antibody SC121 (**Fig 3A**, **3E** and **Supplemental 7**) (see staining details below). After evaluating human cell engraftment, 11 mouse brains were stained for mAb against GFP for transgene expression (**Fig 3A** and **3F**). hNSC-derived myelin/myelinating oligodencytes were tested in 34 homozygous shiverer mouse brains by staining antibody against human MBP, which did not stain for mouse MBP in shiverer brains (**Fig 3G** and **3H**). Three mouse brains were analyzed for Sox2, GFAP and DCX along with GFP (**Fig 3B-D**). There were no differences observed between females and males for human cell engraftment and

transgene expression. All animal housing conditions and surgical procedures were approved by and conducted according to the Institutional Animal Care and Use Committee at StemCells, Inc.

Histology

Transplanted mice were anesthetized and perfused with PBS followed by 4% paraformaldehyde. Brains were serially sectioned (50μm) in the sagittal plane with a freezing microtome (Leica SM2400). For immunoperoxidase staining with SC121 (1:1000, StemCells Inc.), anti-MBP (Millipore 1:500), and anti-GFP (1:500, Invitrogen), brain sections were stained with primary antibodies, followed by biotinylated horse anti-mouse mAb secondary antibodies (1:500, Vector Laboratory). Peroxidase staining was developed with an Elite ABC kit (Vector Laboratory) with NovaRed substrate (Vector Laboratory). Brain sections were mounted and counterstained with methyl green. For confocal immunofluorescence microscopy, imaging was performed on a Leica SP2 AOBS microscope (Leica Microsystems) or a LSM 780 (Zeiss). Brain sections were stained with antibodies against GFP, MBP, Sox 2 (R&D System, 1:200), SC123 (human GFAP, StemCells. Inc 1:3000), and Doublecortin (Santa Cruz 1:200). Secondary antibodies were incubated at RT for 2 hours: Alexa Fluor 488 donkey anti-rabbit (1:500 Invitrogen), Alexa Fluor donkey anti-goat 568 (1:500 Invitrogen), Alexa donkey anti-mouse 568 (1:500, Invitrogen) and counterstained with Hoechest or DAPI to identify nuclei.

Targeted amplicon library generation for MiSeq runs

IL2RG (ON) and the top three *in silico* predicted off target (OFF1-3) Cas9-sgRNA amplicons were PCR-amplified with sequencing primers utilized in deep sequencing MiSeq runs as previously reported(Hendel et al., 2015). Amplicons were gel purified and then subjected to a second round of PCR to add adapters and unique 8bp barcodes to distinguish experiments. Barcoded amplicons were then purified and pooled in equimolar concentrations. The purified library was sequenced on an illumina MiSeq DNA sequencer at 2 x 200 cycles with indexing at the Protein and Nucleic Acid (PAN)

Stanford Core Facility. Sequences were aligned to the human genome and INDELs were calculated as described below.

MiSeq analyses of in silico predicted IL2RG sgRNA off-target sites

IL2RG (ON) and top three *in silico* predicted OFF-target Cas9-sgRNA sites were quantified by mapping reads from each samples to the four amplicon sequence target regions (*IL2RG*, OFF1-3) and measuring number of mapped reads with a gap in a 10 base pair neighborhood of the cut site. Specifically, each read was first assigned to one of the target regions by finding a perfect match between the first 70 bases of each read and the amplicon sequence. If the first 70 base pairs of a read do not perfectly map to any amplicon sequence the read is discarded, resulting in the removal of a large number of low-quality reads from each sample. Reads that do not span a whole amplicon are also discarded. Each read was then aligned with its corresponding target amplicon sequence using EMBOSS version 6.5.7.0 needle with default parameters(Rice et al., 2000). To quantify the number of reads with INDELs, each read was marked as modified if at least one insertion or deletion (a "-" in the alignment of either read or the amplicon) occurs within 5bp up or downstream of the CRISPR cut site (between bases 17 and 18 of the guide RNA sequence). The overall INDEL percentage at a given target site was reported as the number of modified reads mapping to the locus over the total number of reads mapping to the locus, minus the background INDEL percentage in a non-electroporated sample.

GALC enzyme cross-correction of GALC-deficient Krabbe disease Fibroblasts

Fibroblasts from two patients with infantile Krabbe disease were purchased from Coriell Institute for Medical Research (GM06806 and GM08304). Both Krabbe disease fibroblast cell lines have a large deletion in *GALC* that results in deficient GALC enzyme activity (**Supplemental Figure 6C and 6D**), which has been previously reported(Rafi et al., 1995, Luzi et al., 1995). To assess whether GALC-NSCs could rescue GALC activity in Krabbe disease Fibroblasts, we performed cross -correction experiments where GALC-NSCs were co-cultured in 3μm transwell permeable polyester membrane inserts (Corning) with Krabbe fibroblasts. To achieve this, 100,000 fibroblasts were seeded

overnight in NSC media and allowed to adhere and on the following day 1,000,000 GALC-NSCs were added to the permeable membrane insert. Cells were co-cultured for 7 days and half of the fibroblasts were harvested for GALC enzyme activity (passage 1) and the other half were seeded overnight in NSC where on the following day fresh GALC-NSCs were co-cultured in the membrane insert. After 7 more days of cross-correction (and 14 days total), Krabbe fibroblasts were harvested, protein was isolated and GALC enzyme activity was performed as described below. To inhibit GALC enzyme uptake, mannose-6-phosphate (Sigma) was added to the media every 24-48h at a final concentration of 2.5mM.

GALC enzyme assay

Cellular protein was extracted by lysing cells in deionized water with a Branson Sonicator with probe, centrifuging lysates at 17,000xg for 10 minutes at 4°C, and collecting the supernatant containing the soluble proteins. Protein concentration in the supernatants was measured by Bradford assay kit with BSA standard curve ranging from 0.25-.5 mg/ml (Thermo Scientific). To prepare the GALC assay substrate, 250 uL of .9mM 6-hexadecanoyl-4-methylumbelliferyl-beta-D-galactopyranoside (Sigma) in chloroform/methanol (2/1 v/v), 75 uL of 6mg/mL oleic acid in hexane, and 50 uL of 30mg/mL sodium taurocholate in chloroform/methanol (2/1 v/v) were combined and the mixture was dried using a rotary evaporator. The dried mixture was stored under nitrogen at -80 °C covered from light until ready for use. The working substrate was prepared by resuspending the dried substrate in 500 uL assay buffer (50 mM citrate, 100 mM phosphate, pH 5.0) to a final concentration of 0.45 mM and sonicating briefly. 25-50 ug protein extract (50 uL) was mixed with 100 uL of working substrate and incubated for 20 hours at 37 °C covered from light. Reactions were stopped with 200 uL stop buffer (0.2 M glycine/NaOH, 0.2% w/v sodiumdodecyl sulfate, 0.2% w/v Triton X-100, pH 10.7). Fluorescence of 4-methylumbeliferone (4MU) liberated by GALC enzyme cleavage was measured using a Molecular Devices SpectraMax M3 multi-mode microplate reader with SoftMax Pro 5 software at excitation and emission wavelengths of 355 nm and 460 nm,

respectively (top read). A standard curve for 4MU was established using 4MU sodium salt (Sigma) in assay buffer.

BRINKMAN, E. K., CHEN, T., AMENDOLA, M. & VAN STEENSEL, B. 2014. Easy quantitative assessment of genome editing by sequence trace decomposition. *Nucleic Acids Res,* 42**,** e168.

DEVER, D. P., BAK, R. O., REINISCH, A., CAMARENA, J., WASHINGTON, G., NICOLAS, C. E., PAVEL-DINU, M., SAXENA, N., WILKENS, A. B., MANTRI, S., UCHIDA, N., HENDEL, A., NARLA, A., MAJETI, R., WEINBERG, K. I. & PORTEUS, M. H. 2016. CRISPR/Cas9 beta-globin gene targeting in human haematopoietic stem cells. *Nature,* 539**,** 384-389.

HENDEL, A., BAK, R. O., CLARK, J. T., KENNEDY, A. B., RYAN, D. E., ROY, S., STEINFELD, I., LUNSTAD, B. D., KAISER, R. J., WILKENS, A. B., BACCHETTA, R., TSALENKO, A., DELLINGER, D., BRUHN, L. & PORTEUS, M. H. 2015. Chemically modified guide RNAs enhance CRISPR-Cas genome editing in human primary cells. *Nat Biotechnol,* 33**,** 985-9.

HENDEL, A., KILDEBECK, E. J., FINE, E. J., CLARK, J. T., PUNJYA, N., SEBASTIANO, V., BAO, G. & PORTEUS, M. H. 2014. Quantifying genome-editing outcomes at endogenous loci with SMRT sequencing. *Cell Rep,* 7**,** 293-305.

LUZI, P., RAFI, M. A. & WENGER, D. A. 1995. Characterization of the large deletion in the GALC gene found in patients with Krabbe disease. *Hum Mol Genet,* 4**,** 2335-8.

RAFI, M. A., LUZI, P., CHEN, Y. Q. & WENGER, D. A. 1995. A large deletion together with a point mutation in the GALC gene is a common mutant allele in patients with infantile Krabbe disease. *Hum Mol Genet,* 4**,** 1285-9.

RICE, P., LONGDEN, I. & BLEASBY, A. 2000. EMBOSS: the European Molecular Biology Open Software Suite. *Trends Genet,* 16**,** 276-7.

UCHIDA, N., BUCK, D. W., HE, D., REITSMA, M. J., MASEK, M., PHAN, T. V., TSUKAMOTO, A. S., GAGE, F. H. & WEISSMAN, I. L. 2000. Direct isolation of human central nervous system stem cells. *Proc Natl Acad Sci U S A,* 97**,** 14720-5.

UCHIDA, N., CHEN, K., DOHSE, M., HANSEN, K. D., DEAN, J., BUSER, J. R., RIDDLE, A., BEARDSLEY, D. J., WAN, Y., GONG, X., NGUYEN, T., CUMMINGS, B. J., ANDERSON, A. J., TAMAKI, S. J., TSUKAMOTO, A., WEISSMAN, I. L., MATSUMOTO, S. G., SHERMAN, L. S., KROENKE, C. D. & BACK, S. A. 2012. Human neural stem cells induce functional myelination in mice with severe dysmyelination. *Sci Transl Med,* 4**,** 155ra136.

VAKULSKAS, C. A., DEVER, D. P., RETTIG, G. R., TURK, R., JACOBI, A. M., COLLINGWOOD, M. A., BODE, N. M., MCNEILL, M. S., YAN, S., CAMARENA, J., LEE, C. M., PARK, S. H., WIEBKING, V., BAK, R. O., GOMEZ-OSPINA, N., PAVEL-DINU, M., SUN, W., BAO, G., PORTEUS, M. H. & BEHLKE, M. A. 2018. A high-fidelity Cas9 mutant delivered as a ribonucleoprotein complex enables efficient gene editing in human hematopoietic stem and progenitor cells. *Nat Med,* 24**,** 1216-1224.
